# Machine learning integration of multi-modal analytical data for distinguishing abnormal botanical drugs and its application in Guhong injection

**DOI:** 10.1186/s13020-023-00873-y

**Published:** 2024-01-02

**Authors:** Zhu Han, Jiandong Zhao, Yu Tang, Yi Wang

**Affiliations:** 1 Pharmaceutical Informatics Institute, College of Pharmaceutical Sciences, Zhejiang University, Hangzhou, 310058 China; 2 Innovation Institute for Artificial Intelligence in Medicine of Zhejiang University, Hangzhou, 310018 China; 3 National Key Laboratory of Chinese Medicine Modernization, Innovation Center of Yangtze River Delta, Zhejiang University, Jiaxing, 314100 China; 4 Tonghua Guhong Pharmaceutical Co., Ltd., 5099 Jianguo Road, Meihekou, 135099 China

**Keywords:** Botanical drug, Quality evaluation, Multi-modal data, Data fusion, Guhong injection

## Abstract

**Background:**

Determination of batch-to-batch consistency of botanical drugs (BDs) has long been the bottleneck in quality evaluation primarily due to the chemical diversity inherent in BDs. This diversity presents an obstacle to achieving comprehensive standardization for BDs. Basically, a single detection mode likely leads to substandard analysis results as different classes of structures always possess distinct physicochemical properties. Whereas representing a workaround for multi-target standardization using multi-modal data, data processing for information from diverse sources is of great importance for the accuracy of classification.

**Methods:**

In this research, multi-modal data of 78 batches of Guhong injections (GHIs) consisting of 52 normal and 26 abnormal samples were acquired by employing HPLC-UV, -ELSD, and quantitative ^1^H NMR (q^1^HNMR), of which data obtained was then individually used for Pearson correlation coefficient (PCC) calculation and partial least square-discriminant analysis (PLS-DA). Then, a mid-level data fusion method with data containing qualitative and quantitative information to establish a support vector machine (SVM) model for evaluating the batch-to-batch consistency of GHIs.

**Results:**

The resulting outcomes showed that datasets from one detection mode (e.g., data from UV detectors only) are inadequate for accurately assessing the product's quality. The mid-level data fusion strategy for the quality evaluation enabled the classification of normal and abnormal batches of GHIs at 100% accuracy.

**Conclusions:**

A quality assessment strategy was successfully developed by leveraging a mid-level data fusion method for the batch-to-batch consistency evaluation of GHIs. This study highlights the promising utility of data from different detection modes for the quality evaluation of BDs. It also reminds manufacturers and researchers about the advantages of involving data fusion to handle multi-modal data. Especially when done jointly, this strategy can significantly increase the accuracy of product classification and serve as a capable tool for studies of other BDs.

**Supplementary Information:**

The online version contains supplementary material available at 10.1186/s13020-023-00873-y.

## Introduction

According to the report, over 800 botanical investigatory new drug (IND) applications and pre-IND meeting requests have been submitted to nearly every review division of the FDA from 1984 to 2018 [1], and the World Health Organization (WHO) has estimated that perhaps 80% of people are dependent largely on botanical products for their primary health care needs [2]. However, to date, only two botanical drugs (BDs) have been approved by FDA for marketing as prescription drugs. One of the main contributing factors impeding the approval process of botanical products is the chemical complexity, for which metabolomic analysis constantly requires laborious efforts. In addition, BDs purportedly exert therapeutic effects by means of synergistic interactions, so reaching a comprehensive chemical analysis is a prerequisite to ensure the potency of BDs.

Despite the rapid advances in analytical methods, holistic standardization of botanical products continues to be a major challenge, as different types of compounds always encompass distinct physicochemical properties. As a result, the standardization of BDs increasingly entails the combination of analytical techniques featuring different principles to capture botanical constituents to the greatest extent. In practice, LC-based methods, for instance, LC-UV, are the most used approaches by virtue of the relatively abundant instrumentation along with the high sensitivity of UV [3, 4]. A large number of research for the quality evaluation of BDs have been carried out using LC-based methodologies [5–7]. Nonetheless, it is often considered impractical to fulfill comprehensive chemical analysis for BDs as there is a need for identical reference materials (RMs) for the identification of analytes. Basically, BDs integrity increases with the number of markers measured qualitatively and quantitatively. In contrast to LC-based methods, the requirement of identical RMs does not exist in NMR. NMR is regarded as a relatively insensitive method, which possesses more universal detection ability and is capable of performing multi-target analysis on a single sample [8–10]. Owing to the increasing availability of NMR, indeed, there are some reports involving the integration of LC-UV, -MS, and NMR for the standardization of complex matrices [11, 12, 28]. However, from a perspective of the gap between the high demand for healthcare products and the quality consistency of different batches of BDs, the combination of commonly used methods (e.g., LC-based pharmaceutical quality control) and emerging techniques [e.g., quantitative ^1^H NMR (q^1^HNMR)] for providing scientific evidence to the development of BDs is still considered underexplored [27].

On the other hand, concomitant with the combination is the effective processing of experimental datasets generated by different analytical techniques. In the field of quality assessment of BDs, data fusion has proven to be a powerful approach for integrating different kinds of information to assist in the overall understanding of a product. Mid-level data fusion, as one of three level data fusion methods (low, mid, and high), in which multi-type features are often extracted from processed data and fused into a new array to find complementary information [13]. Some studies have utilized the mid-level data fusion strategy for classification. Chang et al. [14] established a mid-level data fusion encompassing GC-FID, UV-Vis, ATR-FT-IR, and HPLC-DAD to produce a better classification result than that from a single technique in the quality assessment of belamcandae rhizome antiviral injection. Zhang et al. [15] collected NIR and MIR spectra, from which the data was organized via low- and mid-level data fusion methods, to rapidly detect the extraction process of a traditional Chinese medicine called Xiao’er Xiaoji Zhike Oral Liquid. Additionally, with the rapid development of artificial intelligence, machine learning algorithms, including but not limited to random forest (RF) [16, 17], support vector machine (SVM) [18], k-nearest neighbor (KNN) [19], have been adopted as powerful tools for feature extraction and data fusion. Of note, previous studies regarding data fusion mainly involved techniques which unveil functional groups of compounds of interest. The present study also applied magnetic examination (e.g., NMR) to provide an alternative for characterizing structures from a point of view of whole molecule.

As a proof of concept, this study developed a quality assessment method that utilizes a mid-level data fusion strategy to integrate data containing qualitative and quantitative information for the standardization of Guhong injection (GHI) that is a botanical drug derived from a sterile aqueous solution made of safflower extract and aceglutamide used for treating ischemic stroke [20, 21]. First, HPLC-UV and -ELSD were used for the qualitative analysis with the help of identical RMs, while q^1^HNMR was applied for the qualitative and quantitative detection of constituents. Notably, some of the constituents (e.g., amino acids) identified in this study by NMR are in large part undetectable to UV and ELSD detectors. Secondly, qualitative features extracted from chromatographic fingerprints and quantitative features obtained from q^1^HNMR were used for Pearson correlation coefficient (PCC) analysis and partial least square-discriminant analysis (PLS-DA). SVM, a typical machine learning approach, was used to solve the problem of binary classification like discrimination of normal and abnormal samples [22]. Finally, both extracted qualitative and quantitative features were organized as a new dataset for SVM modeling. Compared with PCC analysis and PLS-DA of individual features from HPLC-UV, HPLC-ELSD, and q^1^HNMR, SVM with fused features reached a classification accuracy of 100% for classifying normal batches and individually prepared abnormal samples of GHIs. This quality control strategy can be not only regarded as a reliable approach for identifying chemical components and distinguishing abnormal batches of GHIs but also serve as a useful tool for the standardization of other BDs featuring complex matrices.

## Materials and methods

### Reagents

Normal batches of GHIs were provided by Guhong Pharmaceutical Co., Ltd. (Tonghua, China), labeled from N1 to N52 (Additional file 1: Table S1). Half of the normal batches were added with HCl or fructose manually as abnormal batches (also see Additional file 1: Table S2 for details). HPLC-grade solvents were purchased from Merck (Darmstadt, Germany), and methanol-*d*_4_ (99.8 atom %D) with 0.03% (v/v) tetramethylsilane (TMS) was purchased from Cambridge Isotope Laboratories Inc. (Andover, MA, USA). Methyl 3,5-dinitrobenzoate (99.40%) was purchased from Sigma-Aldrich Co.LLC (Switzerland) as the internal calibrant for q^1^HNMR analysis. Chemical reference standards were purchased from Yuanye Biotechnology Co. Ltd (Shanghai, China) and Sigma-Aldrich Co. LLC (Switzerland).

### Sample preparation

#### Sample preparation for HPLC-UV analysis

Guhong injection (1.0 mL) was diluted by 20% methanol (4.0 mL), and centrifuged for 10 min at 10000 rpm·min^−1^. The supernatant was used for HPLC-UV analysis.

#### Sample preparation for HPLC-ELSD analysis

Guhong injection (1.0 mL) was diluted by 70% acetonitrile (4.0 mL), and centrifuged for 10 min at 10000 rpm min^−1^. The supernatant was then used for HPLC-ELSD analysis.

#### Sample preparation for q^1^HNMR analysis

6.03 mg of methyl 3,5-dinitrobenzoate was accurately weighed and dissolved in 10 mL of methanol-*d*_4_. 600 μL of the prepared deuterated solution was added to freeze-dried GHIs, which were transferred into 5 mm NMR tubes for subsequent q^1^HNMR analysis.

### HPLC–UV analysis

Agilent 1100 HPLC system (Agilent Co., USA) equipped with VWD detector was used for HPLC–UV analysis. The chromatographic separation was accomplished with Waters Altantis@T3 column (4.6 × 250 mm, 5 μm), and the mobile phase consisted of 0.1% formic acid (A) and 70% acetonitrile (B). The gradient elution was programmed as follows: 0–12 min, 4% B; 12–20 min, 4–18% B; 20–30 min, 18–19% B; 30–43 min, 19–34% B; 43–47 min, 34–48% B; 47–56 min, 48–100%B. The total run time was 70 min. The flow rate was 0.9 mL·min^−1^, the column temperature was maintained at 35 ℃, and the injection volume was 10 μL.

The HPLC-UV analysis method was validated by precision, repeatability, and stability tests, where the relative standard deviation (RSD) of the average relative retention time (RRT) and relative peak area (RPA) of each characteristic peak with respect to the reference peak were employed for evaluation. To be specific, precision was determined by analyzing the same sample six times. Repeatability was evaluated by the analysis of six parallel prepared samples consecutively. Stability was confirmed by testing the same sample at the time intervals of 0, 3, 6, 12, 18, and 24 h.

### HPLC-ELSD analysis

HPLC-ELSD analysis was performed on an Agilent 1260 HPLC system (Agilent Co., USA). A Prevail Carbohydrate-ES (250 × 4.6 mm, 5 μm) column was used for chromatographic separation. Deionized H_2_O (A) and acetonitrile (B) were used as mobile phases. The gradient was as follows: 0–25 min, 88–85% B; 25–45 min, 85–70% B; 45–49 min, 70–60% B; 49–50 min, 60–50% B and 50–55 min, 50% B. The flow rate was set at 0.7 mL min^−1^ and the column temperature was set at 35 °C. The evaporator temperature was 60 °C and the nebulizer temperature was 50 °C for ELSD, respectively. The nitrogen flow rate was set as 1.2 L min^−1^ and the gain value was 1.

Method validation was accomplished by investigating precision, repeatability, and stability. Precision was estimated by six consecutive injections of a sample. Repeatability was also evaluated by analyzing six parallel prepared samples consecutively. Stability was assessed by testing the same sample at the time points of 0, 4, 8, 12, 20, and 24 h.

### q^1^HNMR analysis

q^1^HNMR analysis was performed on JEOL ECZ-500 (Akishima, Tokyo, Japan). Automatic shimming and adjusting 90° pulse length before each sample acquisition. The ^1^HNMR spectra parameters were set as follows: the number of scans was 16, acquisition time (AQ) was 3.2 s, and pulse width was 20.0 ppm. To ensure fully quantitative conditions for the target signals, the relaxation delay time (D_1_) was set to 60.0 s.

The q^1^HNMR spectra were phase-adjusted, baseline-corrected, and unified to TMS at 0.000 ppm using MestReNova 14.0.0 software from Mestrelab Research S.L. (Santiago de Compostela, Spain). Due to the complex chemical composition and additives’ interference of injections that caused the overlap of spectra peaks, eight main characteristic peaks were selected for quantification. The obtained peak areas were utilized to calculate the content of the components according to Eq. (1) [23].

1 $$C_{x} { = }\frac{{N_{IC} * A_{x} * M_{x} }}{{N_{x} * A_{IC} * M_{IC} }} * C_{IC}$$

where *N* represents the number of integrated hydrogens, *A* is the absolute integral value, *M* is the molar mass, *C* represents the mass concentration, *IC* is the internal calibrant, and *x* is the target analyte or molecule.

### Feature processing

**Fig. 1:**
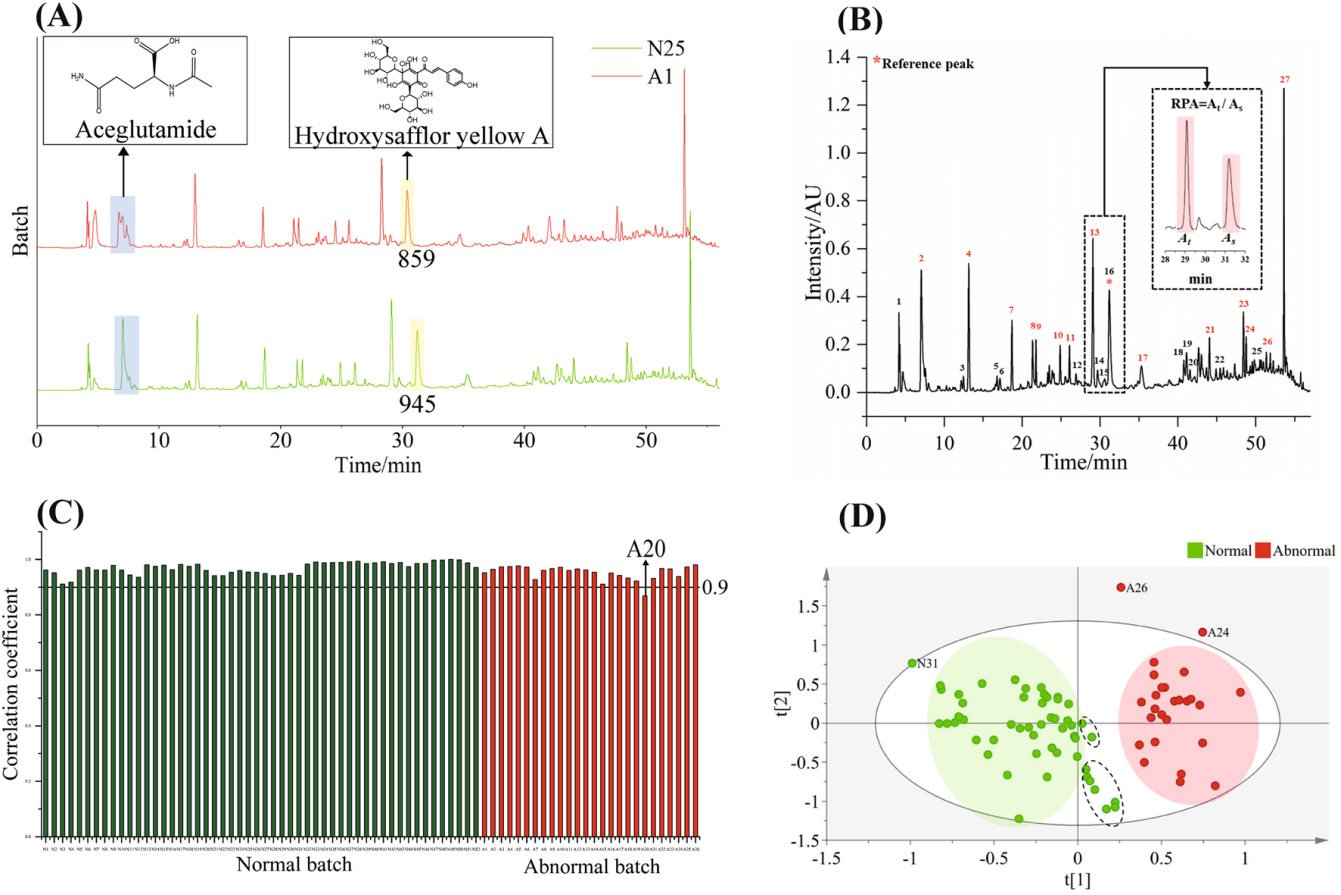
HPLC–UV data analysis. **A** Comparison of aceglutamide (peak 2) and hydroxysafflor yellow A (peak 16) between normal (N25) and abnormal (A1) batches, the data showed that the peak shape of peak 2 and the absolute integral values of peak 16 were significantly changed. **B** Peak 16 was used as the reference peak and relative peak area (RPA) was calculated as the value of the AUC of the target peak (A_t_) dividing by the AUC of the reference peak (A_s_). **C** Pearson correlation coefficient between batches was analyzed by involving the RPA values calculated. It showed that only sample A20 was recognized as the abnormal sample by setting the threshold value at 0.9. **D** PLS-DA using the RPA values: nine normal samples (in the dashed ellipse) were out of the cluster (green area) along with one normal (N31) and two abnormal (A24 and A26) samples falling outside of the 95% confidence interval

**Fig. 2:**
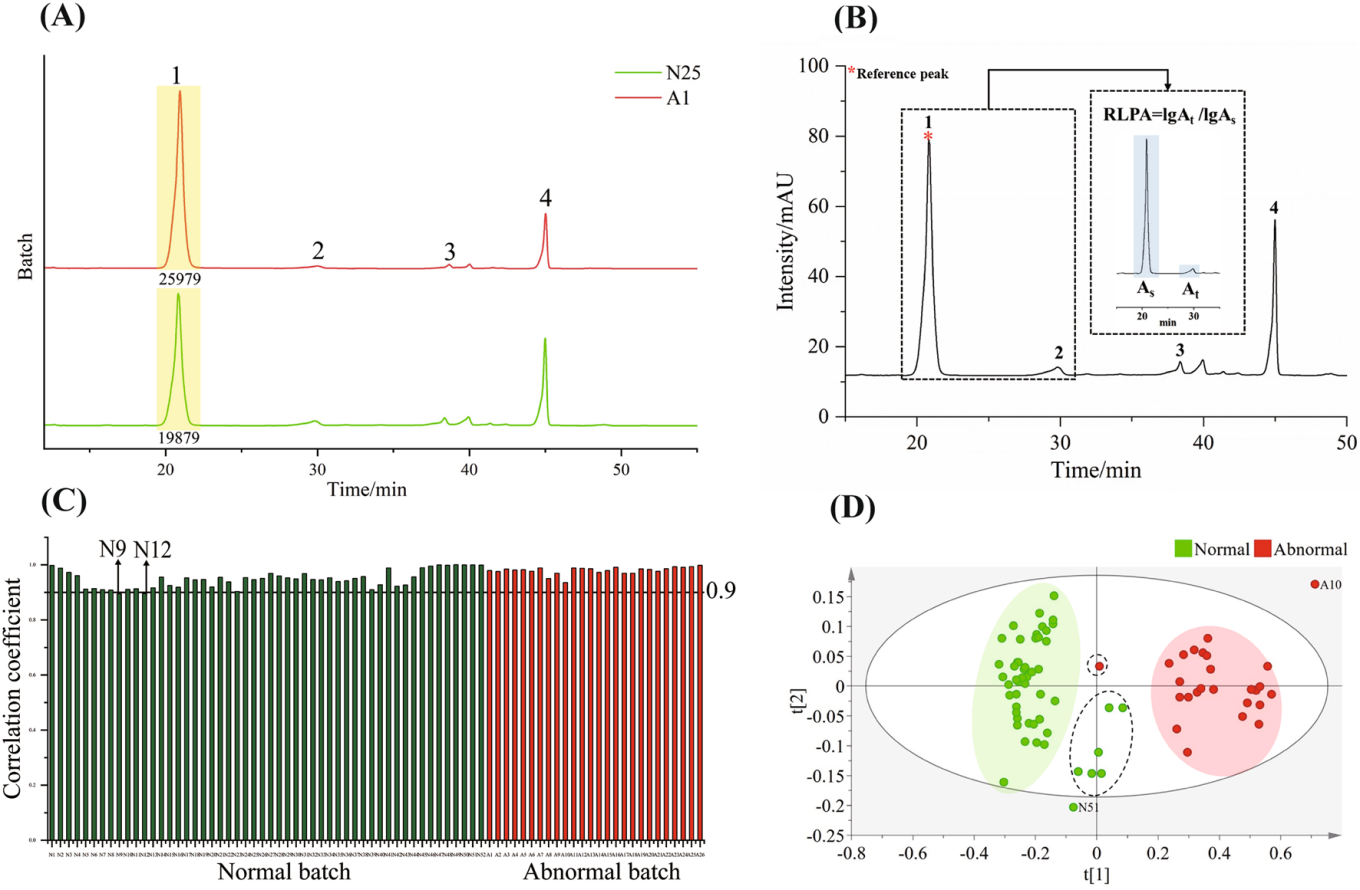
HPLC-ELSD data analysis. **A** HPLC-ELSD profiles of the normal and abnormal samples along with the sugars (peak 1 and peak 2) and glycosides (peak 3 and peak 4) identified. **B** Peak 1 was selected as the reference peak and RLPA was calculated as the value of the AUC of the target peak (A_t_) dividing by the AUC of the reference peak (A_s_). **C** Pearson correlation coefficient between batches was analyzed using the RLPA values. Two normal samples (N9 and N12) were characterized unexpectedly less similar to both normal and abnormal samples. **D** PLS-DA of the ELSD data using the RLPA values. One abnormal and six normal samples (in the dashed ellipses) were out of the individual clusters along with normal sample (N51) and abnormal sample (A10) falling outside of the 95% confidence interval

RPA was calculated by dividing the area under the curve (AUC) of the target peak by the AUC of the reference peak in HPLC-UV chromatograms, while the log value of the relative peak area (RLPA) was obtained by dividing the log value of the AUC of the target peak by the log value of the AUC of the reference peak (see Figs. 1 and 2 and the text for details). RPA and RLPA from each batch were separately utilized for creating qualitative feature tables for subsequent analysis and modeling.

**Fig. 3:**
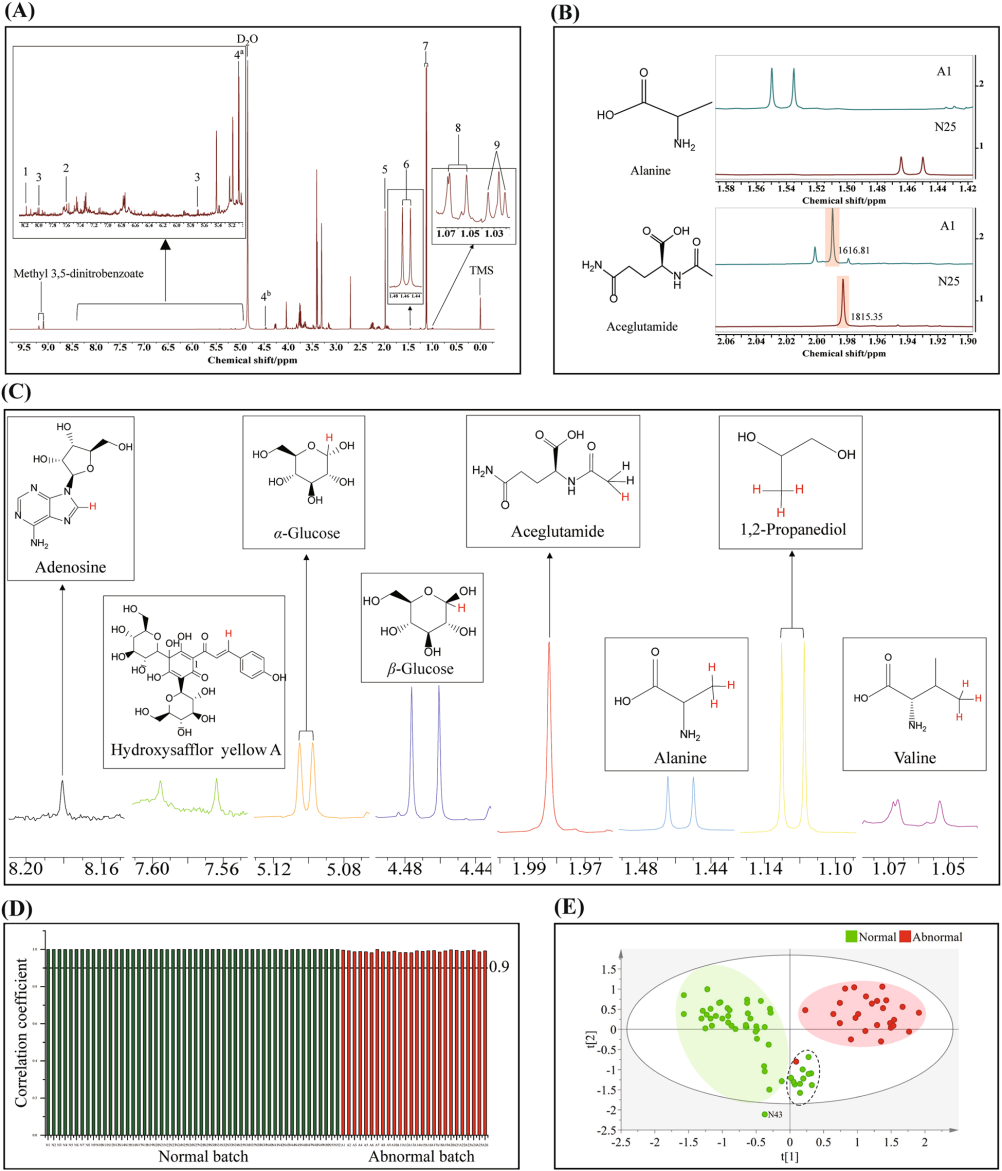
q^1^HNMR data analysis of the normal and abnormal samples. **A** The representative q^1^HNMR spectrum of GHI and characteristic signals selected. In total, nine compounds were identified (also see Table 1 for details). **B** Comparison of chemical shifts changing and occurrence of unexpected peaks of aceglutamide and alanine between the normal and abnormal batches. **C** Among the identified compounds, seven of them were selected for further content determination as a result of the availability of well resolved signals for quantification. **D** Pearson correlation coefficient between 78 batches was analyzed by using the content values obtained from q^1^HNMR data. The results indicated that all the samples were showed excellent similarities when setting the threshold value at 0.9. **E** PLS-DA was employed for classifying the normal and abnormal samples based on the q^1^HNMR data. One abnormal and eleven normal samples were out of the individual clusters along with one normal sample (N43) falling outside of the 95% confidence interval

The effective chemical shift ranges of each q^1^HNMR spectrum were determined after phase adjusting and baseline correction. Compounds were characterized based on 1&2D NMR in conjunction with reference standards. The characteristic peaks (see Table 1 for details) of identified compounds were used for the content calculation by an internal standard method (see Fig. 3, Table 1, and the text for details). The content calculated of the identified compounds from each batch was tabulated for further analysis and modeling.

**Table 1:** Characterized compounds by q^1^HNMR and their characteristic signals

No	Chemical compounds	*δ*_H_ (multiplicity, *J* in Hz)
1^*^	Adenosine	8.18 (s)
2^*^	Hydroxysafflor yellow A	7.58 (d, *J* = 15.8 Hz)
3	Uridine	5.70 (d, *J* = 7.9 Hz), 8.01 (d, *J* = 7.9 Hz)
4^a*^	*α*-Glucose	5.10 (d, *J* = 3.7 Hz)
4^b*^	*β*-Glucose	4.47 (d, *J* = 7.8 Hz)
5^*^	Aceglutamide	1.98 (s)
6^*^	Alanine	1.46 (d, *J* = 7.2 Hz)
7^*^	1,2-Propanediol	1.13 (d, *J* = 6.3 Hz)
8^*^	Valine	1.06 (d, *J* = 7.0 Hz)
9	Isoleucine	1.03 (d, *J* = 6.9 Hz)

^*^Components quantified

### PCC analysis and PLS-DA

In this study, RPA, RLPA, and the content of characterized compounds were separately used for PCC analysis to determine the similarity between each batch.

2 $$r = \frac{{\sum\limits_{i = 1}^{N} {({\rm A}_{i} } - \overline{\rm A}) * ({\rm B}_{i} - \overline{\rm B})}}{{\sqrt {\sum\limits_{i = 1}^{N} {({\rm A}_{i} } - \overline{\rm A})^{2} } * \sqrt {\sum\limits_{i = 1}^{N} {({\rm B}_{i} - \overline{\rm B})^{2} } } }}$$

*r* represents Pearson correlation coefficient,* A*_*i*_ represents the reference value and *B*_*i*_ represents the target value, $\overline{A}$ and $\overline{B}$ represent the mean value of the standards and target compounds, respectively.

Apart from PCC analysis, RPA, RLPA, and the content of characterized compounds of each batch were also input into SIMCA software (version 14.1, MKS Umetrics AB, Umeå, Sweden) to accomplish PLS-DA to evaluate the classification effect of different types between batches from an overall perspective.

### Mid-level data fusion

**Scheme 1:**
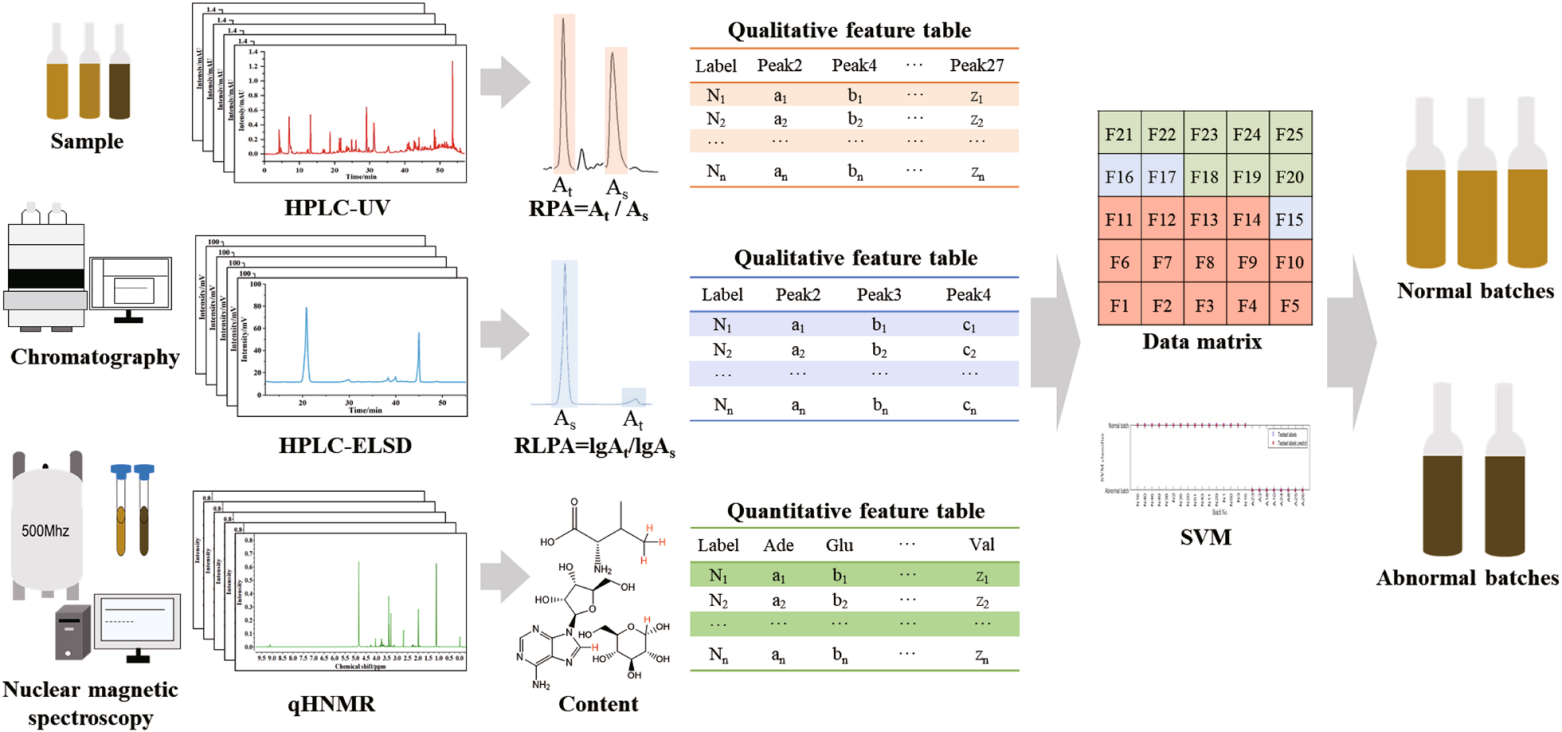
Summary of the mid-level data fusion strategy for the classification of GHIs. After acquiring multi-modal data, features were extracted from those data acquired and fused as a dataset for SVM modeling to differentiate GHIs

In this study, a mid-level data fusion strategy was established aimed at improving classification accuracy for distinguishing abnormal batches. The strategy of feature extraction and mid-level data fusion was summarized in Scheme 1. First of all, multi-batch samples were prepared and analyzed by HPLC-UV, HPLC-ELSD, and NMR to obtain chromatographic fingerprints and nuclear magnetic spectra. Second, RPA and RLPA as qualitative features of HPLC-UV and -ELSD, respectively, along with content of compounds identified as quantitative features of q^1^HNMR, were extracted for creating feature tables. Third, the qualitative and quantitative features of each batch were fused as a new data matrix for subsequent modeling analysis. Machine learning is an advisable choice to enhance the efficiency of data processing and the accuracy of classification. Moreover, SVM, a classic machine learning method, is commonly used for dealing with the problem of binary classification. Collectively, SVM was applied in this study to deal with the multi-modal data for a more accurate quality evaluation of BDs. The SVM model was established by MATLAB (Version: R2023a). All input data was taken by data normalization, and radial basis function was applied for training the SVM classifier. Tenfold cross-validation was used for the model validation and a hyperparameter was found to minimize tenfold cross-validation losses by using automatic hyperparameter optimization.

## Results and discussion

### HPLC-UV analysis

In our previous study, the HPLC-UV fingerprint of GHI was established with a total of 27 peaks labeled, among them, 26 ones were identified by LC-MS [21]. As a continuous work for the standardization of BDs, the present study acquired the HPLC-UV chromatograms (Additional file 1: Fig. S1) of another 52 normal and 26 in-house developed abnormal batches of GHIs according to the previously established method. As shown in Fig. 1A, the peak shape of peak 2 in the abnormal batch was distorted, while the AUC of peak 16 significantly changed between the normal and abnormal batches with a relative deviation of 9.12%. In addition, peak 16, which stands for the main bioactive marker of GHI, namely hydroxysafflor yellow A, showed good separation from other peaks, appropriate signal intensity, and reasonable retention time, and was hence selected as the reference peak for RPA calculation. Accordingly, RPA was defined as the value of the AUC of the selected peak (A_t_) over the AUC of the reference peak (A_s_) as shown in Fig. 1B. Taking into account the degree of separation and the signal intensity, 14 peaks of peak 2, 4, 7, 8, 9, 10, 11, 13, 17, 21, 23, 24, 26, and 27 were determined for creating a qualitative feature table of the HPLC-UV data of the 78 batches (Additional file 1: Table S3), which were used for the subsequent analysis including PCC analysis and PLS-DA. Similarity evaluation of the HPLC-UV data was conducted by the PCC analysis and RPA of N49 was randomly chosen as the reference dataset for calculating the PCC values (Additional file 1: Table S4). With 0.9 set as the threshold value, only A20 was identified as an abnormal one among the 26 abnormal samples (Fig. 1C). Then, PLS-DA was used for evaluating the class of samples with the RPA values as the dependent and the sample types as independent variables as shown in Fig. 1D. Nine normal samples among the two dashed ellipses were not well clustered with the majority of other normal samples. Meanwhile, N31, A26, and A24 were clustered out of the 95% confidence interval. Besides, a permutation test was involved for validating the validity of PLS-DA (Additional file 1: Fig. S2). To confirm the reliability of HPLC-UV fingerprints, method validation was completed by precision, repeatability, and stability analysis, the results were described in supplement information (method validation of HPLC-UV fingerprints).

### HPLC-ELSD analysis

In this work, four UV-transparent compounds in GHIs were detected by HPLC-ELSD and identified as fructos (peak 1), glucose (peak 2), gycerol-1-*O*-galactfpyr-anosyl-(1 → 4)-*O*-ara- binofuranoside (peak 3), and glycerol-1-*O*-galactpyranosyl-(1 → 4)[*O*-ara-binfuranosyl -(1 → 3)]-*O*-ara-binofuranoside (peak 4) referenced to the literature [24]. The newly acquired HPLC-ELSD chromatograms of the 78 samples were stacked as shown in Additional file 1: Fig. S3. Although the peak shape showed visually no difference, the AUC disclosed considerable difference as evidenced by peak 1 (25979 of A1 *vs* 19879 of N25). The RLPA were obtained by dividing the log values of the AUC of the target peaks (peaks 2, 3, 4) with the log value of the AUC of the reference peak (peak1, Fig. 2B). All the calculated RLPA values were tabulated (Additional file 1: Table S5) and then used for the PCC analysis and PLS-DA. Still, the dataset of N49 was used as the reference and the PCC results (Fig. 2C and Additional file 1: Table S6) revealed that N9 and N12 were below the threshold of 0.90. As to the PLS-DA (Fig. 2D), One abnormal and six normal batches among the dashed ellipses were not well classified into the corresponding classes. N51 and A10 were beyond the 95% confidence interval. The permutation test was utilized for validating the validity of PLS-DA as shown in Additional file 1: Fig. S4. Details of method validation were shown in supplement information (method validation of HPLC–UV fingerprints).

### q^1^HNMR analysis

As a versatile and judicious detector, in this study, q^1^HNMR was used as well for the multi-component characterization of the 78 samples (Fig. 3A and Additional file 1: Fig. S5). Nine compounds, including adenosine, uridine, hydroxysafflor yellow A, aceglutamide, 1,2-propanediol, glucose, alanine, valine, and isoleucine, were identified according to the data reported in the literature [25, 26], in conjunction with the 2D NMR spectra (Additional file 1: Figs. S6, S7, S8, and S9) and the q^1^HNMR of reference standards (Additional file 1: Fig. S10).

The differences observed in the q^1^HNMR spectra were shown in Fig. 3B exemplified by sample N25 and A1: (i) the appearance of a singlet (1.98 ppm) near the methyl group of aceglutamide in the abnormal sample indicated the occurrence of degradation; (ii) the chemical shift of the methyl group of alanine is up field shifted from 1.46 to 1.54 ppm. This might be caused by the protonation of the amino groups in these two components as a result of the addition of HCl. On the other hand, the characteristic signals of the identified nine compounds were summarized in Table 1, among them, the content of seven ones (Fig. 3C) in the 78 samples, except uridine and isoleucine due to intense signal overlap, was determined through an internal calibration q^1^HNMR method with methyl 3,5-dinitrobenzoate used as the internal standard (Additional file 1: Table S7). The deconvolution results for resolving signal overlap of valine for quantitation (Additional file 1: Fig. S11). Subsequently, the PCC analysis (Fig. 3D) and PLS-DA (Fig. 3E) were performed based on the calculated content (Additional file 1: Table S8). Clearly, the PCC results can not differentiate the normal and abnormal samples at the threshold value of 0.9, while in total twelve samples including one normal and eleven abnormal ones in the dashed ellipse were not classified into the corresponding clusters according to the PLS-DA. The permutation test is also used to determine the validity of PLS-DA (Additional file 1: Fig. S12).

### Mid-level data fusion

**Fig. 4:**
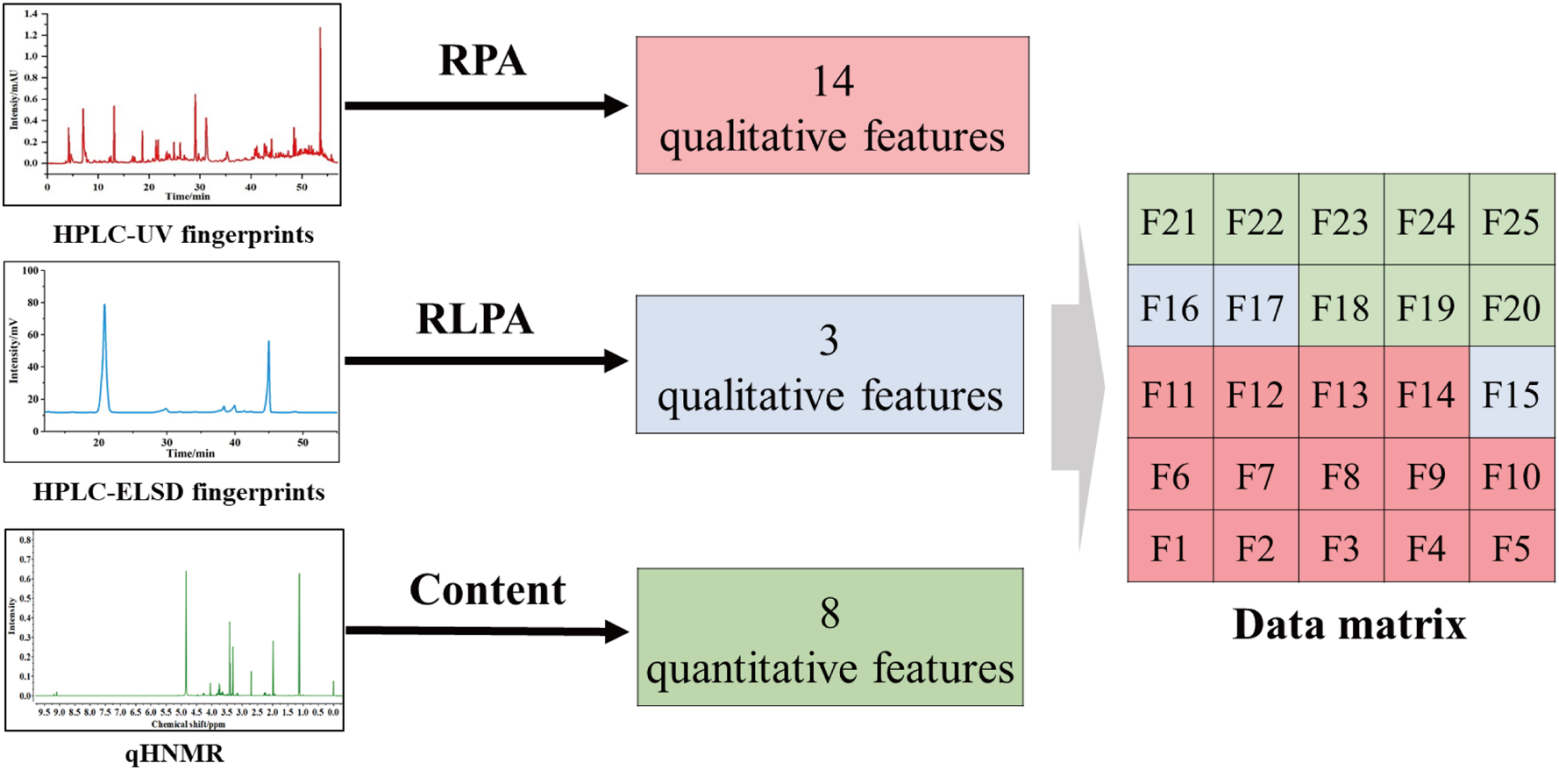
Feature extraction and fusion flowchart of the multi-modal data. 14 qualitative features of RPA were extracted from HPLC-UV fingerprints, 3 qualitative features of RLPA were extracted from HPLC-ELSD fingerprints and 8 quantitative features of components’ content were extracted from q^1^HNMR. These features were fused as a new data matrix for subsequent model analysis

**Fig. 5:**
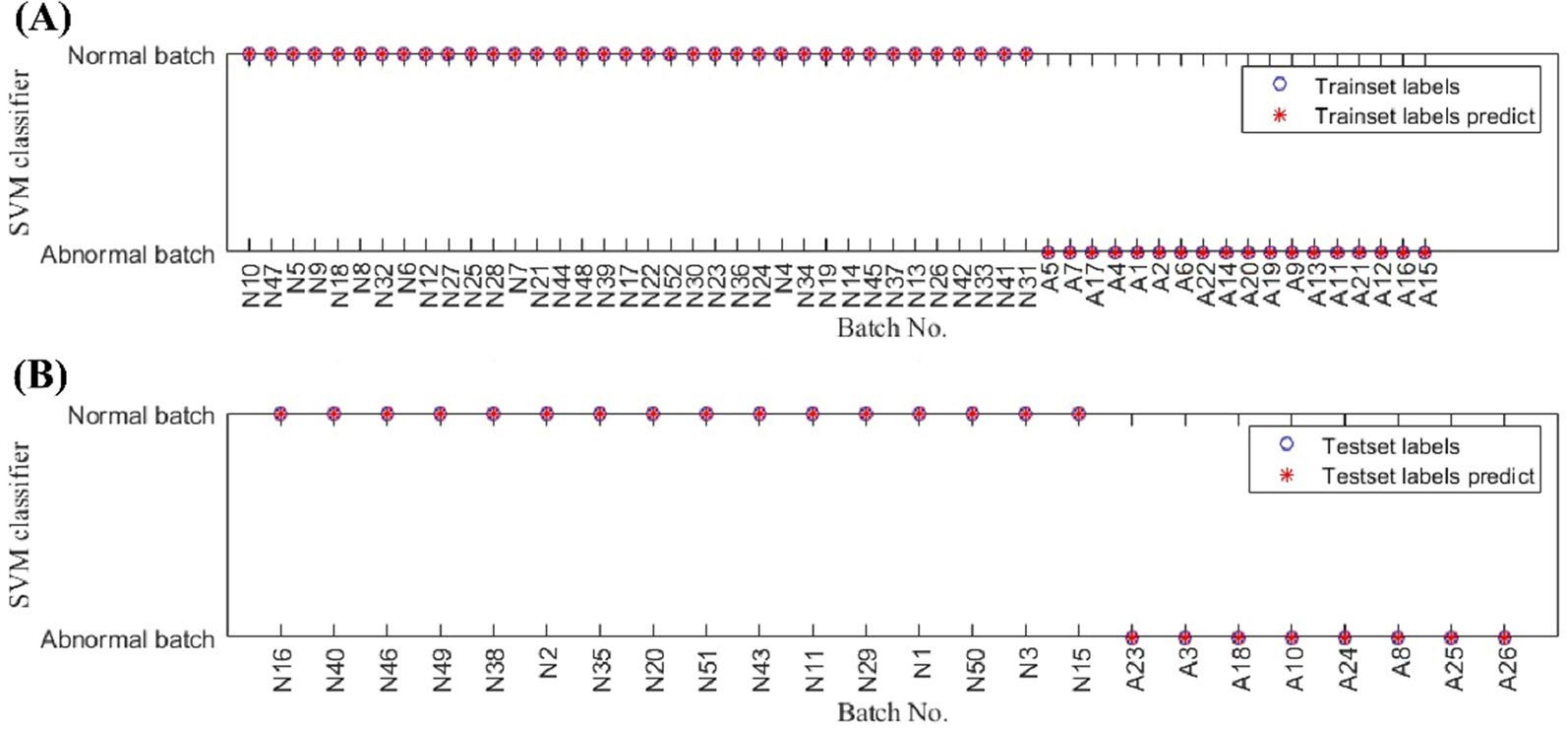
SVM model for classifying normal and abnormal batches based on mid-level data fusion. **A** 36 normal and 18 abnormal samples were used as the trainset for training SVM model. **B** 16 normal and 8 abnormal samples were used as the test set to verify the robustness of the model

In the mid-level data fusion strategy, 14 qualitative features of RPA from HPLC-UV fingerprints, 3 qualitative features of RLPA from HPLC-ELSD fingerprints, and 8 quantitative features of components’ content from q^1^HNMR were fused as a new data matrix as shown in Fig. 4. The fused data table of all 78 batches were showed in the Additional file 1: Table S9. The batch number of 52 normal batches and 26 abnormal batches was randomly sorted. 70 and 30% of the whole dataset were respectively utilized for the train set and test set, respectively (Additional file 1: Table S10). The SVM model results of the trainset are shown in Fig. 5A, in which blue circle represents the trainset label and the red start represents prediction trainset label. Basically, if the red dot is located in the blue circle, the batch is precisely classified into the correct category. According to Fig. 5A, both normal and abnormal batch samples were all sorted into the corresponding class, indicating that the classification accuracy was 100% for the trainset.

In order to verify the applicability of the established SVM model, we then applied this model to evaluate the test set. The results demonstrated that the classification accuracy was also 100% for the test set as shown in Fig. 5B. Compared with the PCC analysis and PLS-DA for individual features from HPLC-UV, HPLC-ELSD, and q^1^HNMR, the mid-level data fusion with SVM revealed better classification performance. According to the accurate classification of normal and abnormal batch samples of GHIs based on the data fusion strategy, we quickly distinguished abnormal batches for better quality control of products.

## Conclusions

The present study demonstrated that mid-level data fusion integrating multi-type datasets and comprehensive chemical characterization is currently the most capable strategy to enable accurate quality evaluation of complex samples such as BDs. Using clinical GHIs as the study materials, the present work realized an identification of normal and abnormal batches of the injections at 100% accuracy by employing a data fusion workflow that encompasses the following key steps: (i) RPA and RLPA as qualitative features were extracted from the HPLC-UV and -ELSD fingerprints, respectively; (ii) content of the identified compounds by q^1^HNMR as quantitative feature were calculated. Subsequently, (iii) qualitative and quantitative features were utilized individually for the similarity analysis and PLS-DA to evaluate the discrepancy between different batches. Then, (iv) qualitative and quantitative features were fused as a new data matrix for SVM modeling. The mid-level data fusion with SVM was successfully applied for classifying normal and abnormal batches with an accuracy of 100%. This quality control strategy can be not only regarded as a reliable approach for identifying chemical components and distinguishing abnormal batches of GHIs but also serve as a useful tool for the standardization of other BDs featuring complex matrices.

### Supplementary Information

**Additional file 1: Figure S1.** Chromatographic fingerprints of 78 batches of GHIs based on HPLC-UV. **Figure S2.** The permutation test to validate the validity of PLS-DA based on RPA of HPLC-UV fingerprints. **Figure S3.** Chromatographic fingerprints of 78 batches of GHIs based on HPLC-ELSD. **Figure S4.** The permutation test to validate the validity of PLS-DA based on RLPA of HPLC-ELSD fingerprints. **Figure S5.** q1HNMR spectra of 78 batches of GHIs. **Figure S6.** 13C NMR spectrum of GHI in CD3OD. **Figure S7.** HSQC spectrum of GHI in CD3OD. **Figure S8.** COSY spectrum of GHI in CD3OD. **Figure S9.** HMBC spectrum of GHI in CD3OD. **Figure. S10.** Comparisons between the spectra of reference compounds and Guhong Injection. **Figure. S11.** Deconvolution resolved signal overlap for q1HNMR quantitation of Valine. **Figure S12.** The permutation test to validate the validity of PLS-DA based on the content of compounds by q1HNMR. **Table S1.** Label, Lot number and Volume of 52 normal batches of GHIs. **Table S2.** Label, Lot number and Volume of 26 abnormal batches of GHIs. **Table S3.** RPA extracted from HPLC-UV fingerprints of 78 batches of GHIs. **Table S4.** Pearson correlation coefficient based on RPA between 78 batches of GHIs. **Table S5.** RLPA extracted from HPLC-ELSD fingerprints of 78 batches of GHIs. **Table S6.** Pearson correlation coefficient based on RLPA between 78 batches of GHIs. **Table S7.** Absolute concentrations of compounds from 78 batches of GHIs (mg·mL-1). **Table S8.** Pearson correlation coefficient based on content between 78 batches of GHIs. **Table S9.** Fused feature table. **Table S10.** Trainset based on fused feature table. **Table S11.** Testset based on fused feature table. **Method validation S1.** Method validation of HPLC-UV fingerprints. **Method validation S2.** Method validation of HPLC-ELSD fingerprints.

## Data Availability

The datasets used and/or analyzed among the current study are available from the corresponding author on reasonable request.

## Abbreviations

BDs: Botanical drugs
GHIs: Guhong injections
PCC: Pearson correlation coefficient
q^1^HNMR: Quantitative ^1^H NMR
LC-MS: Liquid chromatography-mass spectrometry
PLS-DA: Partial least square-discriminant analysis
IND: Investigatory new drug
WHO: World Health Organization
RMs: Reference materials
SVM: Support vector machine
RF: Random forest
TMS: Tetramethylsilane
KNN: K-nearest neighbor
RRT: Relative retention time
RSD: Relative standard deviation
RPA: Relative peak area
AUC: Area under the curve
RLPA: Log value of relative peak area
