# Supplementary material for: Machine learning integration of multi-modal analytical data for distinguishing abnormal botanical drugs and its application in Guhong injection
Source: Chin Med. 2024 Jan 2;19:2. doi: 10.1186/s13020-023-00873-y (PMC10759515; doi:10.1186/s13020-023-00873-y)
Supplement: Supplementary file 1 — Additional file 1: Figure S1. Chromatographic fingerprints of 78 batches of GHIs based on HPLC-UV. Figure S2. The permutation test to validate the validity of PLS-DA based on RPA of HPLC-UV fingerprints. Figure S3. Chromatographic fingerprints of 78 batches of GHIs based on HPLC-ELSD. Figure S4. The permutation test to validate the validity of PLS-DA based on RLPA of HPLC-ELSD fingerprints. Figure S5. q1HNMR spectra of 78 batches of GHIs. Figure S6. 13C NMR spectrum of GHI in CD3OD. Figure S7. HSQC spectrum of GHI in CD3OD. Figure S8. COSY spectrum of GHI in CD3OD. Figure S9. HMBC spectrum of GHI in CD3OD. Figure. S10. Comparisons between the spectra of reference compounds and Guhong Injection. Figure. S11. Deconvolution resolved signal overlap for q1HNMR quantitation of Valine. Figure S12. The permutation test to validate the validity of PLS-DA based on the content of compounds by q1HNMR. Table S1. Label, Lot number and Volume of 52 normal batches of GHIs. Table S2. Label, Lot number and Volume of 26 abnormal batches of GHIs. Table S3. RPA extracted from HPLC-UV fingerprints of 78 batches of GHIs. Table S4. Pearson correlation coefficient based on RPA between 78 batches of GHIs. Table S5. RLPA extracted from HPLC-ELSD fingerprints of 78 batches of GHIs. Table S6. Pearson correlation coefficient based on RLPA between 78 batches of GHIs. Table S7. Absolute concentrations of compounds from 78 batches of GHIs (mg·mL-1). Table S8. Pearson correlation coefficient based on content between 78 batches of GHIs. Table S9. Fused feature table. Table S10. Trainset based on fused feature table. Table S11. Testset based on fused feature table. Method validation S1. Method validation of HPLC-UV fingerprints. Method validation S2. Method validation of HPLC-ELSD fingerprints. [file 13020_2023_873_MOESM1_ESM.docx]

**Machine learning integration of multi-modal analytical data for distinguishing abnormal botanical drugs and its application in Guhong Injection**

Zhu Han^a^, Jiandong Zhao ^d^, Yu Tang^a,c^*, Yi Wang^a,b,c^*

^a^ Pharmaceutical Informatics Institute, College of Pharmaceutical Sciences, Zhejiang University, Hangzhou 310058, China

^b^ Innovation Institute for Artificial Intelligence in Medicine of Zhejiang University, Hangzhou 310018, China

^c^ National Key Laboratory of Chinese Medicine Modernization, Innovation Center of Yangtze River Delta, Zhejiang University, 314100, Jiaxing, China

^d^ Tonghua Guhong Pharmaceutical Co., Ltd., 5099 Jianguo Road, Meihekou 135099, China

* Corresponding Author: [yutang@zju.edu.cn](mailto:yutang@zju.edu.cn); [zjuwangyi@zju.edu.cn](mailto:zjuwangyi@zju.edu.cn)

**Supplementary Information**

**Content**

[Supplemental Figures 3](#_Toc9827)

[Figure. S1. Chromatographic fingerprints of 78 batches of GHIs based on HPLC-UV 3](#_Toc17487)

[Figure. S2. The permutation test to validate the validity of PLS-DA based on RPA of HPLC-UV fingerprints 4](#_Toc29649)

[Figure. S3. Chromatographic fingerprints of 78 batches of GHIs based on HPLC-ELSD 5](#_Toc4904)

[Figure. S4. The permutation test to validate the validity of PLS-DA based on RLPA of HPLC-ELSD fingerprints 6](#_Toc3709)

[Figure. S5. q^1^HNMR spectra of 78 batches of GHIs 7](#_Toc10138)

[Figure. S6. ^13^C NMR spectrum of GHI in CD_3_OD 8](#_Toc13664)

[Figure. S7. HSQC spectrum of GHI in CD_3_OD 9](#_Toc20842)

[Figure. S8. COSY spectrum of GHI in CD_3_OD 10](#_Toc31533)

[Figure. S9. HMBC spectrum of GHI in CD_3_OD 11](#_Toc23156)

[Figure. S10. Comparisons between the spectra of reference compounds and Guhong Injection 12](#_Toc25694)

[Figure. S11. Deconvolution resolved signal overlap for q^1^HNMR quantitation of Valine 13](#_Toc22292)

[Figure. S12. The permutation test to validate the validity of PLS-DA based on the content of compounds by q^1^HNMR 14](#_Toc30336)

[Supplemental Tables 16](#_Toc30399)

[Table S1. Label, Lot number and Volume of 52 normal batches of GHIs 16](#_Toc19183)

[Table S2. Label, Lot number and Volume of 26 abnormal batches of GHIs 17](#_Toc15946)

[Table S3. RPA extracted from HPLC-UV fingerprints of 78 batches of GHIs 18](#_Toc2147)

[Table S4. Pearson correlation coefficient based on RPA between 78 batches of GHIs 20](#_Toc8648)

[Table S5. RLPA extracted from HPLC-ELSD fingerprints of 78 batches of GHIs 21](#_Toc511)

[Table S6. Pearson correlation coefficient based on RLPA between 78 batches of GHIs 22](#_Toc25047)

[Table S7. Absolute concentrations of compounds from 78 batches of GHIs (mg·mL^-1^) 23](#_Toc28611)

[Table S8. Pearson correlation coefficient based on content between 78 batches of GHIs 27](#_Toc28887)

[Table S9. Fused feature table 28](#_Toc4999)

[Table S10. Trainset based on fused feature table 32](#_Toc1636)

[Table S11. Testset based on fused feature table 35](#_Toc30450)

[Supplemental Method validation 36](#_Toc31748)

[Method validation S1. Method validation of HPLC-UV fingerprints 36](#_Toc4619)

[Method validation S2. Method validation of HPLC-ELSD fingerprints 36](#_Toc9198)

**Supplemental Figures**


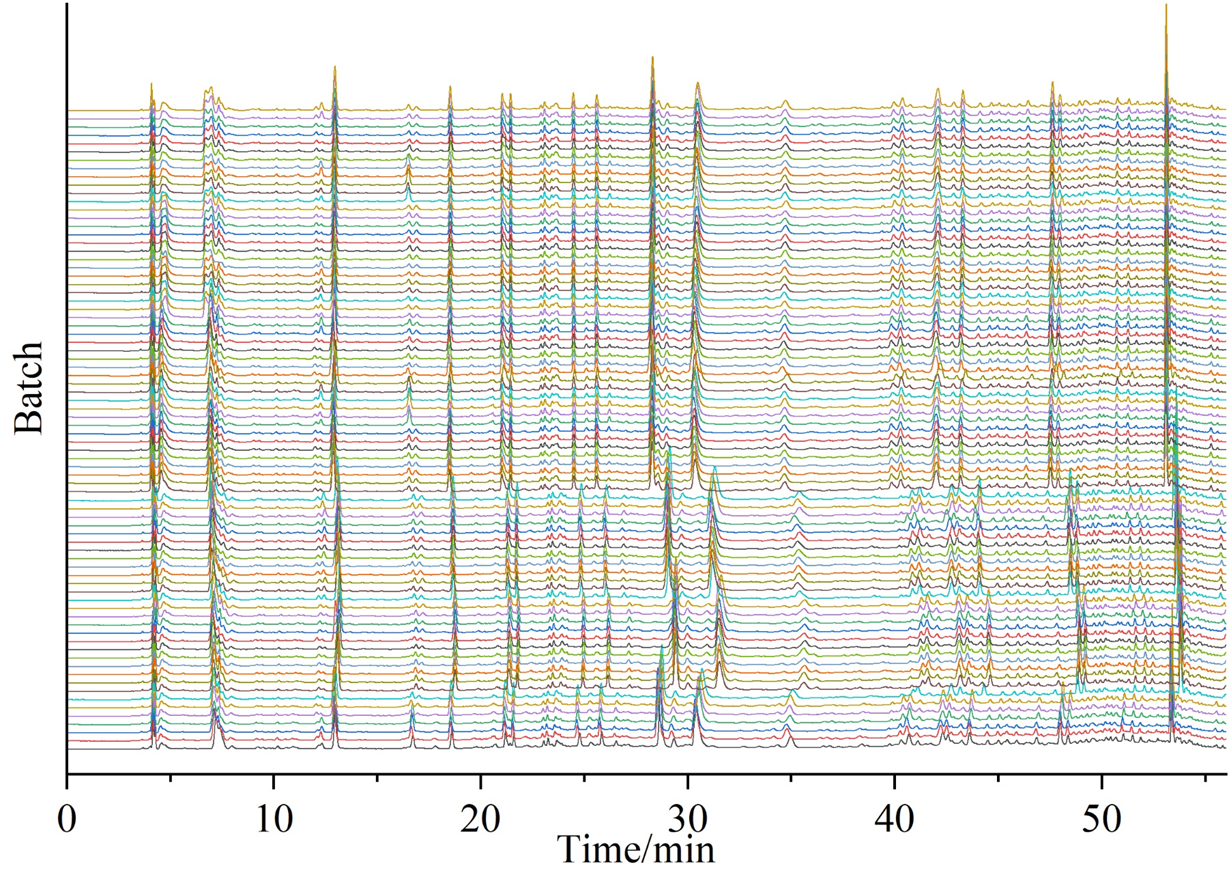


**Figure. S1.** Chromatographic fingerprints of 78 batches of GHIs based on HPLC-UV


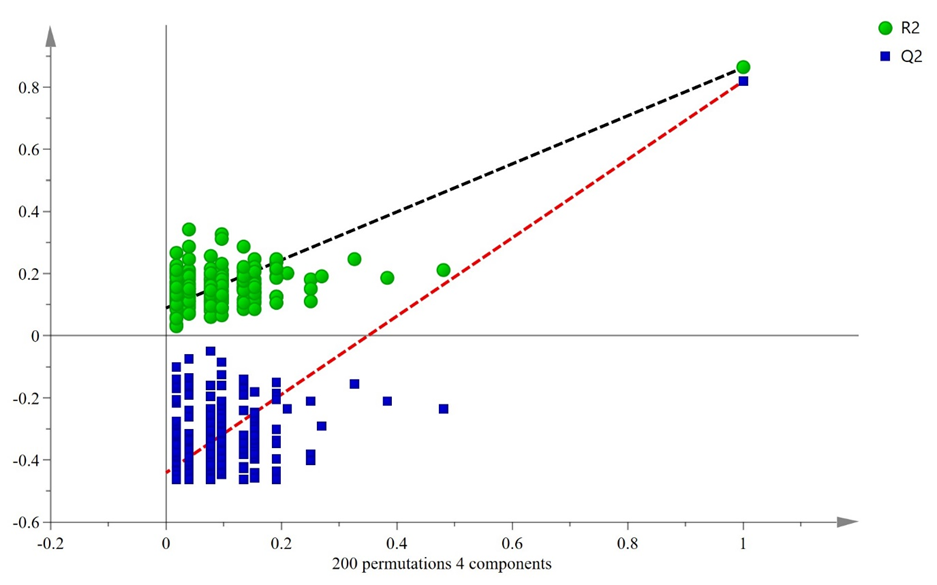


**Figure. S2.** The permutation test to validate the validity of PLS-DA based on RPA of HPLC-UV fingerprints

(The intersection point between the regression line of Q2 (red line) and the vertical axis is less than 0, indicating that there is no over-fitting of the model and the model is effective.)


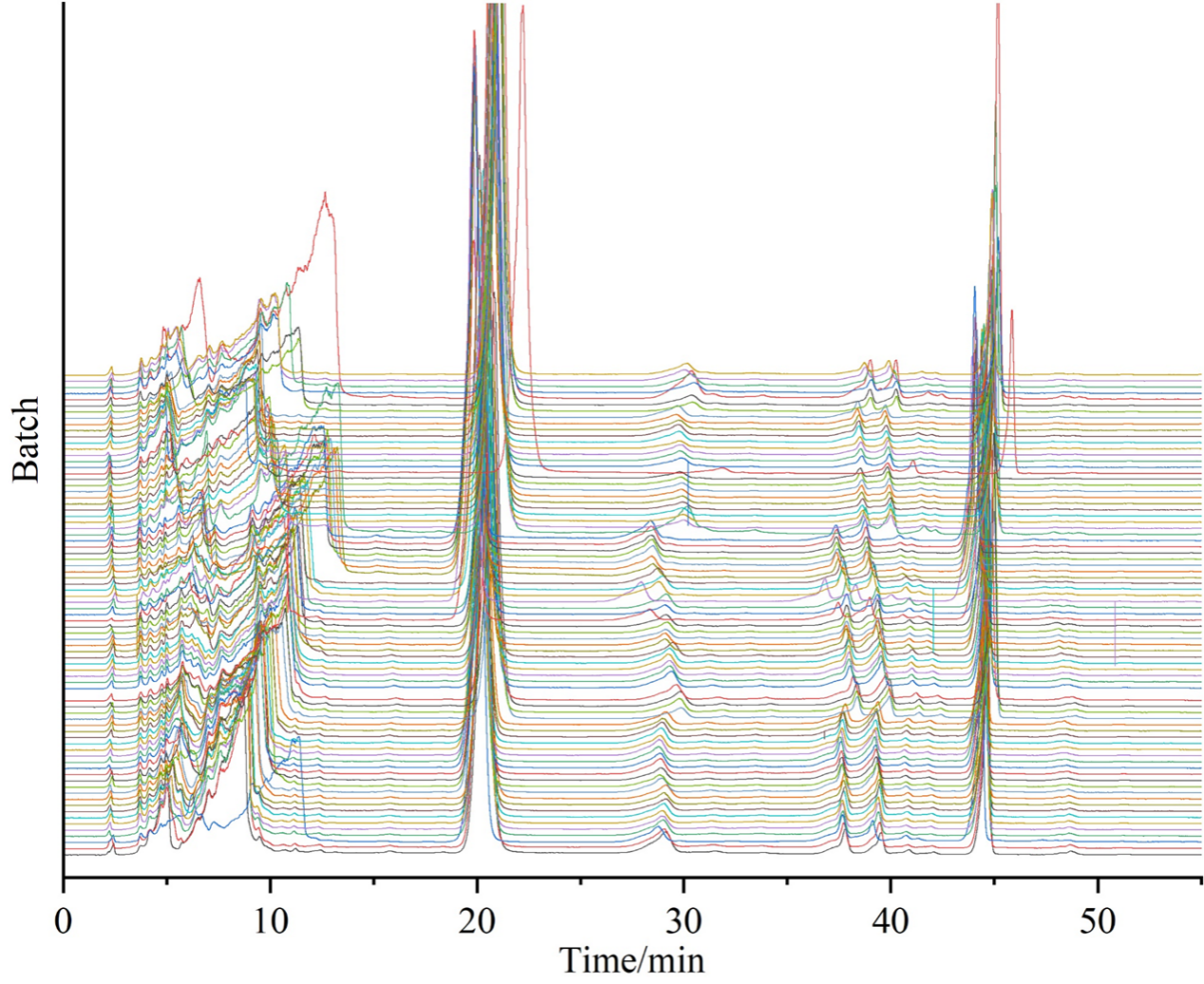


**Figure. S3.** Chromatographic fingerprints of 78 batches of GHIs based on HPLC-ELSD


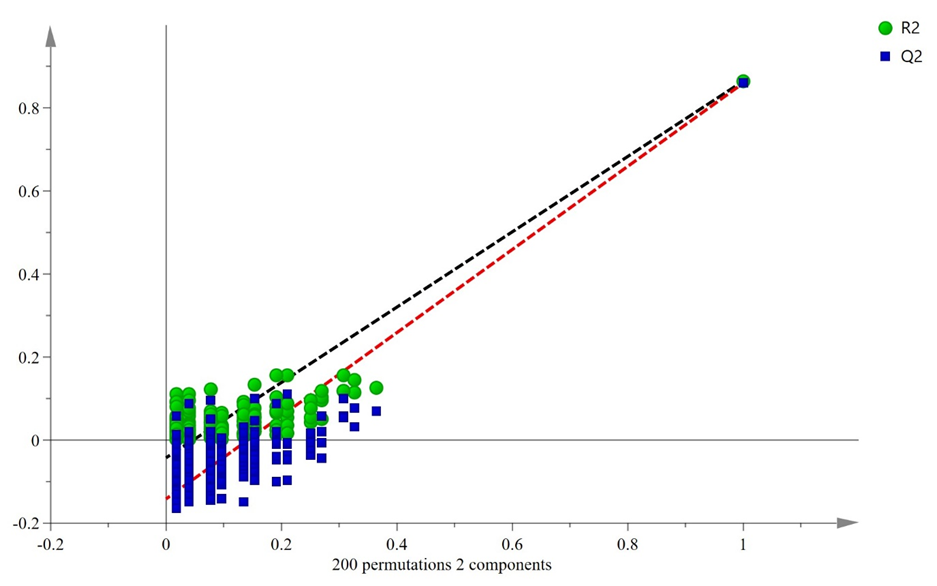


**Figure. S4.** The permutation test to validate the validity of PLS-DA based on RLPA of HPLC-ELSD fingerprints

(The intersection point between the regression line of Q2 (red line) and the vertical axis is less than 0, indicating that there is no over-fitting of the model and the model is effective.)


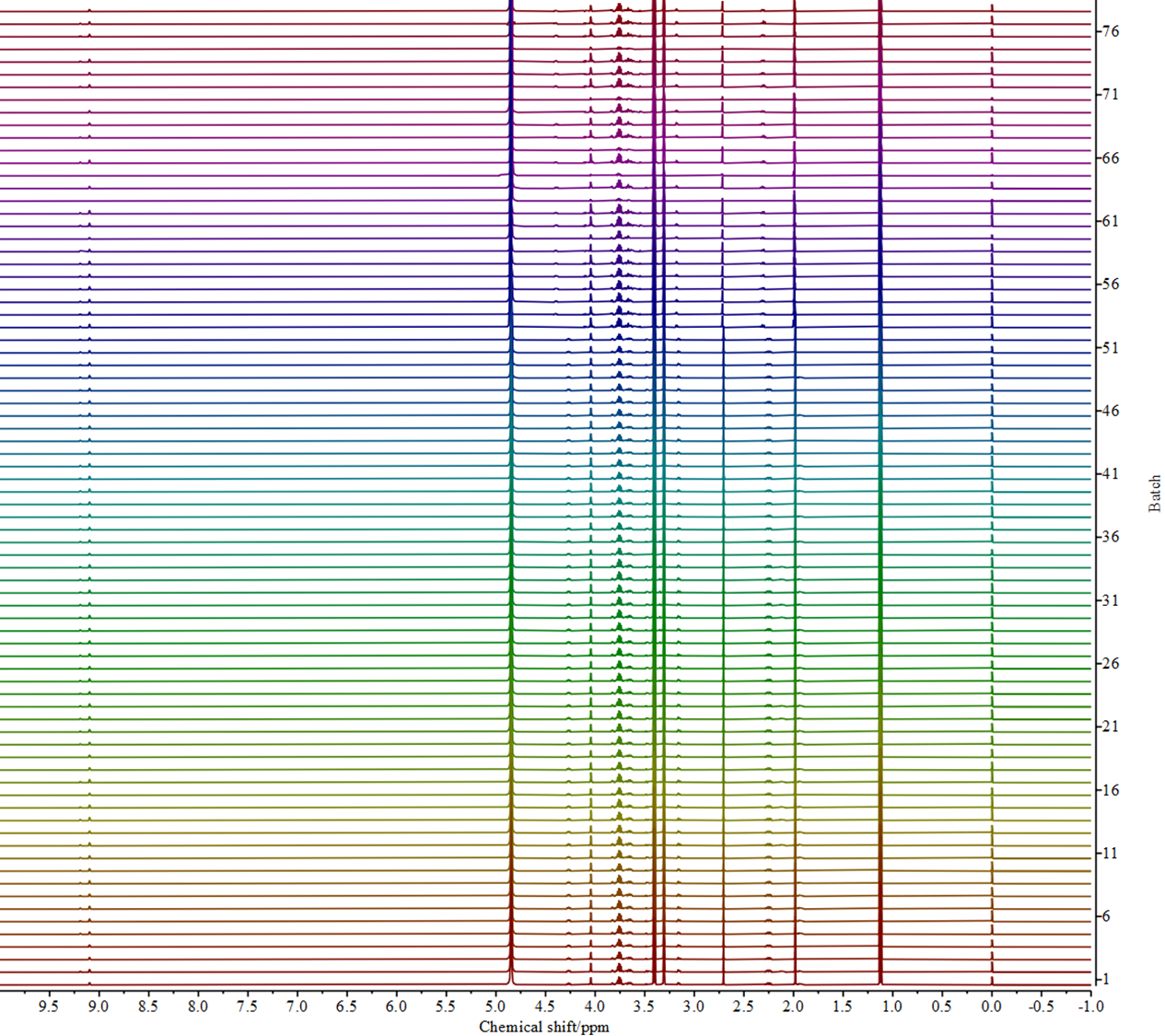


**Figure. S5.** q^1^HNMR spectra of 78 batches of GHIs


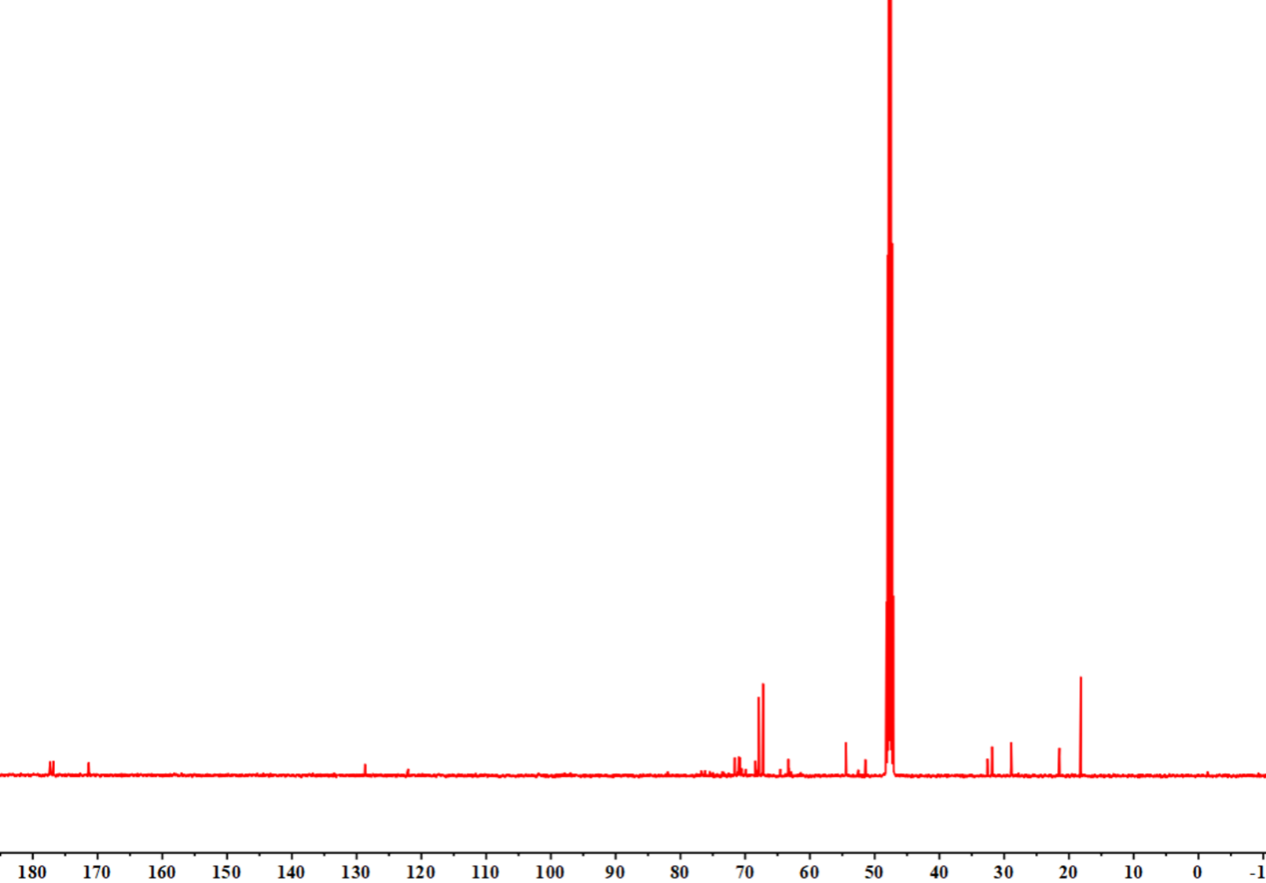


**Figure. S6.** ^13^C NMR spectrum of GHI in CD_3_OD


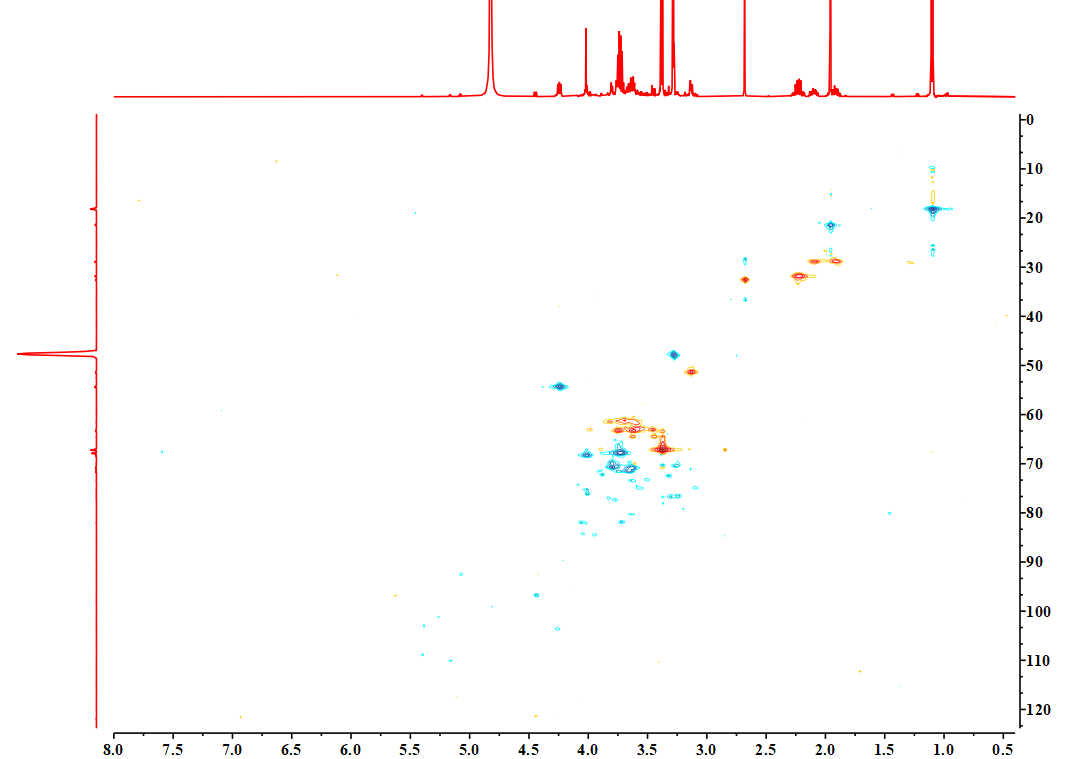


**Figure. S7.** HSQC spectrum of GHI in CD_3_OD


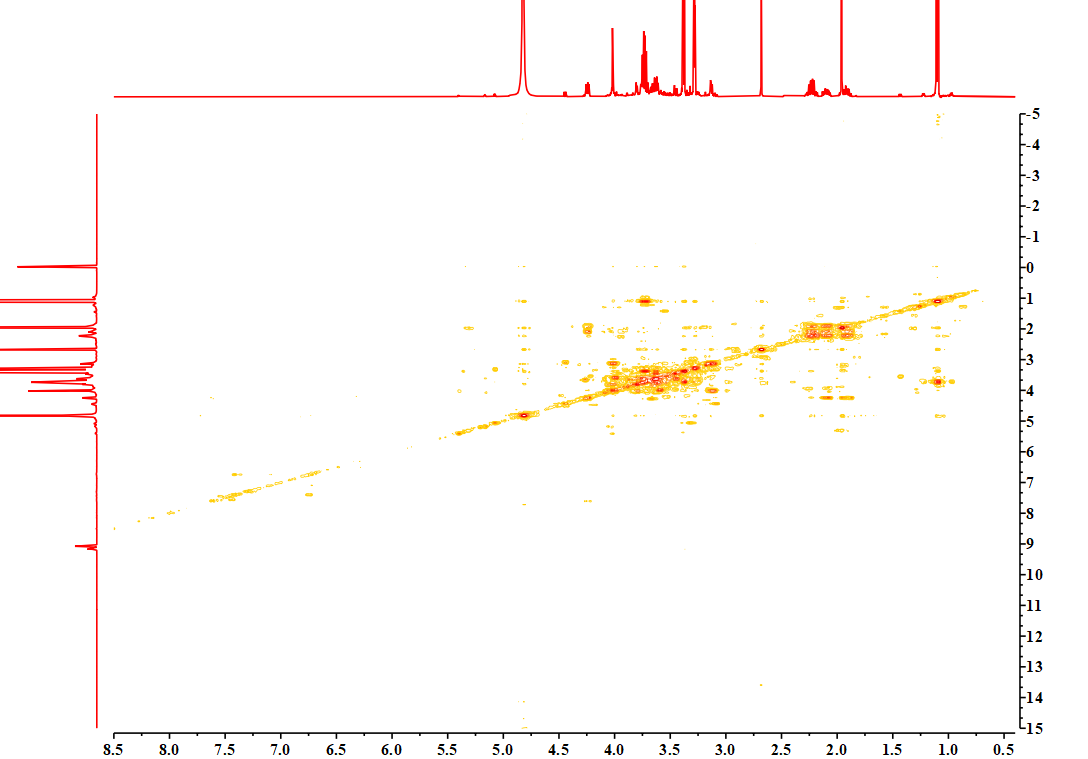


**Figure. S8.** COSY spectrum of GHI in CD_3_OD


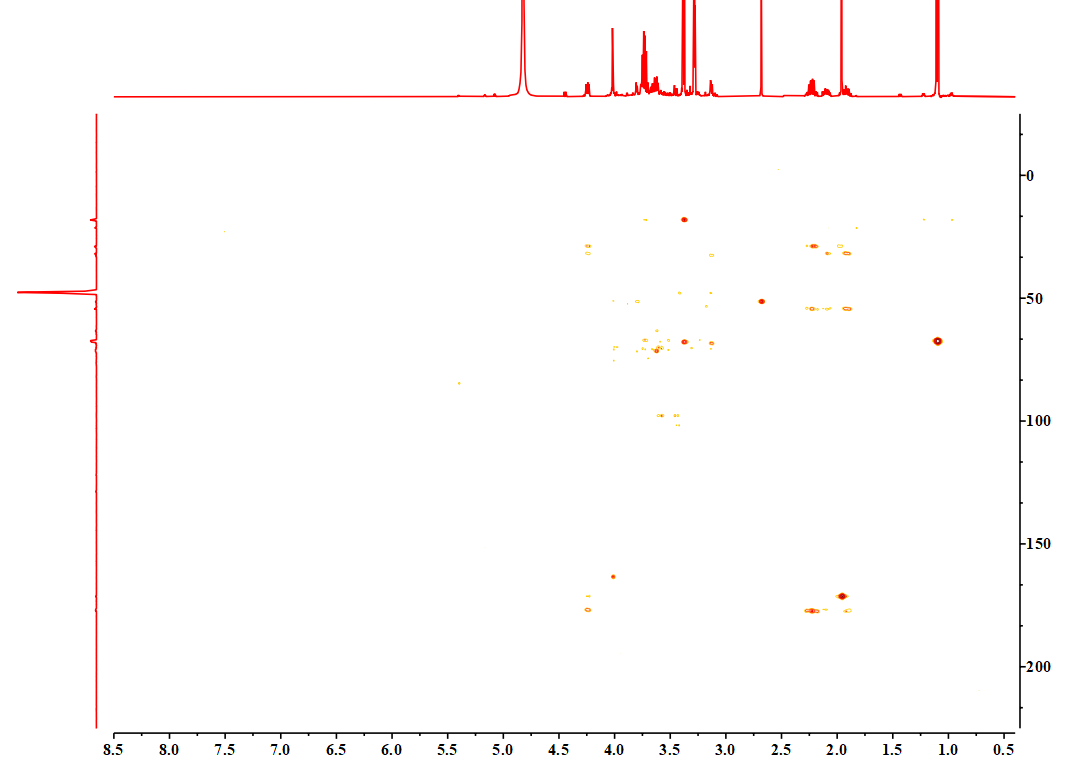


**Figure. S9.** HMBC spectrum of GHI in CD_3_OD


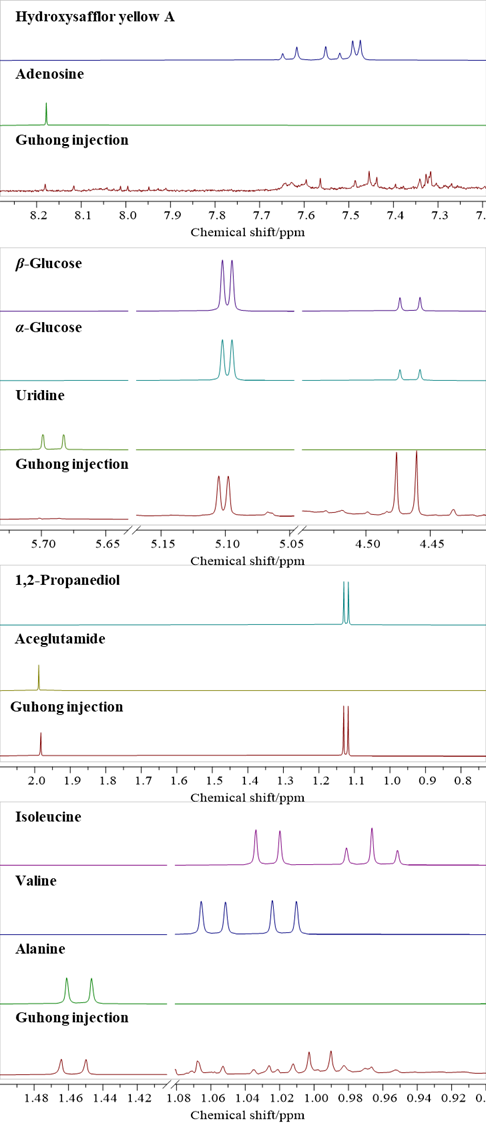


**Figure. S10.** Comparisons between the spectra of reference compounds and Guhong Injection


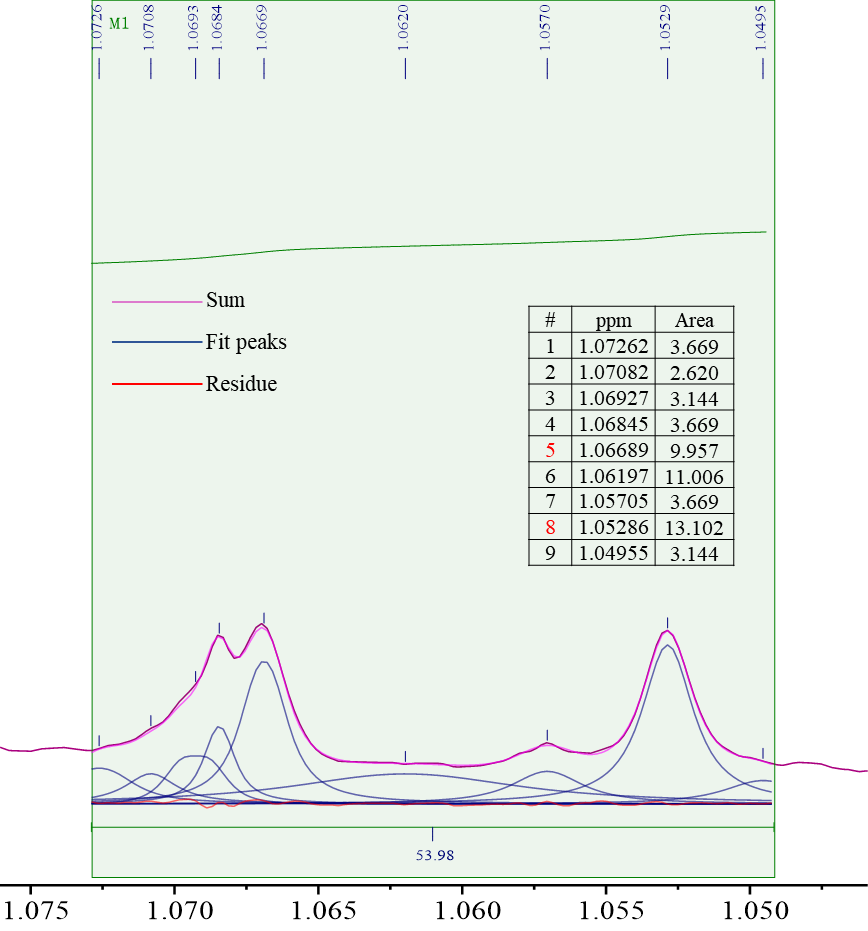


**Figure. S11.** Deconvolution resolved signal overlap for q^1^HNMR quantitation of Valine


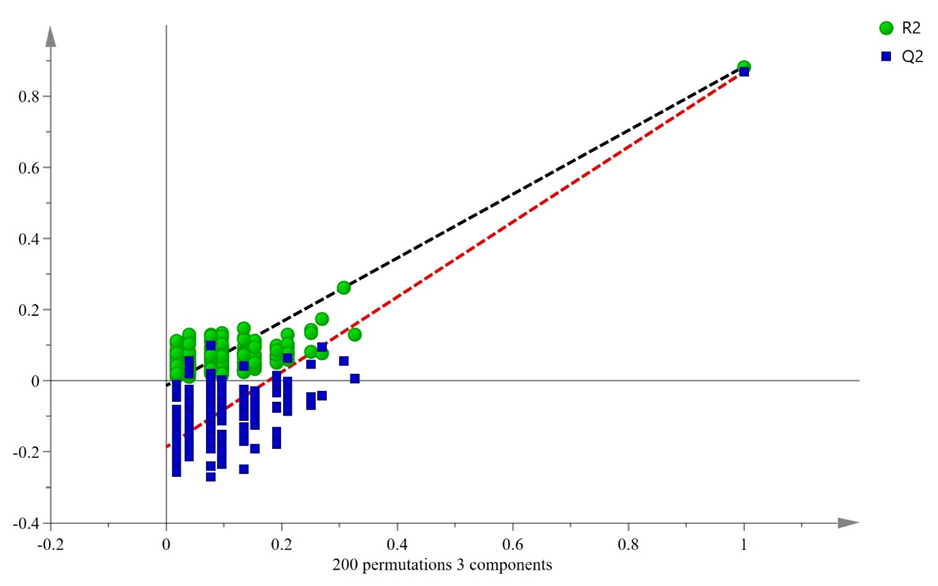


**Figure. S12.** The permutation test to validate the validity of PLS-DA based on the content of compounds by q^1^HNMR

(The intersection point between the regression line of Q2 and the vertical axis is less than 0, indicating that there is no over-fitting of the model and the model is effective.)

**Supplemental Tables**

**Table S1.** Label, Lot number and Volume of 52 normal batches of GHIs

| Label | Lot Number | Volume/mL | Label | Lot Number | Volume/mL |
| --- | --- | --- | --- | --- | --- |
| N1 | GH20210314 | 5 | N27 | GH20221008 | 5 |
| N2 | GH20210724 | 5 | N28 | GH20221009 | 5 |
| N3 | GH20211002 | 5 | N29 | GH20221010 | 5 |
| N4 | GH20211012 | 5 | N30 | GH20221101 | 5 |
| N5 | GH20220603 | 5 | N31 | GH20221102 | 5 |
| N6 | GH20220801 | 5 | N32 | GH20221201 | 5 |
| N7 | GH20220802 | 5 | N33 | GH20221202 | 5 |
| N8 | GH20220803 | 5 | N34 | GH20221203 | 5 |
| N9 | GH20220804 | 5 | N35 | GH20221204 | 5 |
| N10 | GH20220805 | 5 | N36 | GH20221205 | 5 |
| N11 | GH20220806 | 5 | N37 | GH20221206 | 5 |
| N12 | GH20220807 | 5 | N38 | GH20221207 | 5 |
| N13 | GH20220808 | 5 | N39 | GH20221208 | 5 |
| N14 | GH20220809 | 5 | N40 | GH20210112 | 10 |
| N15 | GH20220812 | 5 | N41 | GH20210304 | 10 |
| N16 | GH20220813 | 5 | N42 | GH20210506 | 10 |
| N17 | GH20220814 | 5 | N43 | GH20210706 | 10 |
| N18 | GH20220816 | 5 | N44 | GH20211002 | 10 |
| N19 | GH20220902 | 5 | N45 | GH20220801 | 10 |
| N20 | GH20221001 | 5 | N46 | GH20220802 | 10 |
| N21 | GH20221002 | 5 | N47 | GH20220901 | 10 |
| N22 | GH20221003 | 5 | N48 | GH20220902 | 10 |
| N23 | GH20221004 | 5 | N49 | GH20220903 | 10 |
| N24 | GH20221005 | 5 | N50 | GH20220904 | 10 |
| N25 | GH20221006 | 5 | N51 | GH20221002 | 10 |
| N26 | GH20221007 | 5 | N52 | GH20221003 | 10 |

**Table S2.** Label, Lot number and Volume of 26 abnormal batches of GHIs

| Label | Lot Number for HPLC-UV  and NMR* | Lot Number for HPLC-ELSD** | Volume/mL |
| --- | --- | --- | --- |
| A1 | GH20221006-P | GH20221006-Y | 5 |
| A2 | GH20221007-P | GH20221007-Y | 5 |
| A3 | GH20221008-P | GH20221008-Y | 5 |
| A4 | GH20221009-P | GH20221009-Y | 5 |
| A5 | GH20221010-P | GH20221010-Y | 5 |
| A6 | GH20221101-P | GH20221101-Y | 5 |
| A7 | GH20221102-P | GH20221102-Y | 5 |
| A8 | GH20221201-P | GH20221201-Y | 5 |
| A9 | GH20221202-P | GH20221202-Y | 5 |
| A10 | GH20221203-P | GH20221203-Y | 5 |
| A11 | GH20221204-P | GH20221204-Y | 5 |
| A12 | GH20221205-P | GH20221205-Y | 5 |
| A13 | GH20221206-P | GH20221206-Y | 5 |
| A14 | GH20221208-P | GH20221208-Y | 5 |
| A15 | GH20210112-P | GH20210112-Y | 10 |
| A16 | GH20210304-P | GH20210304-Y | 10 |
| A17 | GH20210506-P | GH20210506-Y | 10 |
| A18 | GH20210706-P | GH20210706-Y | 10 |
| A19 | GH20211002-P | GH20211002-Y | 10 |
| A20 | GH20220801-P | GH20220801-Y | 10 |
| A21 | GH20220802-P | GH20220802-Y | 10 |
| A22 | GH20220902-P | GH20220902-Y | 10 |
| A23 | GH20220903-P | GH20220903-Y | 10 |
| A24 | GH20220904-P | GH20220904-Y | 10 |
| A25 | GH20221002-P | GH20221002-Y | 10 |
| A26 | GH20221003-P | GH20221003-Y | 10 |

* HCl was added into every normal batch to decrease the pH from 5.4 to 1.5 in order to simulate the abnormal batch for HPLC-UV and qHNMR analysis.

** 0.2 mg fructose was added into every normal batch in order to simulate the abnormal batch for HPLC-ELSD analysis.

**Table S3.** RPA extracted from HPLC-UV fingerprints of 78 batches of GHIs

| Label | Peak 2 | Peak 4 | Peak 7 | Peak 8 | Peak 9 | Peak 10 | Peak 11 | Peak 13 | Peak 17 | Peak 21 | Peak 23 | Peak 24 | Peak 26 | Peak 27 |
| --- | --- | --- | --- | --- | --- | --- | --- | --- | --- | --- | --- | --- | --- | --- |
| N1 | 0.78 | 0.48 | 0.19 | 0.19 | 0.13 | 0.22 | 0.21 | 0.84 | 0.27 | 0.30 | 0.33 | 0.16 | 0.22 | 0.75 |
| N2 | 0.58 | 0.63 | 0.24 | 0.29 | 0.18 | 0.33 | 0.28 | 1.07 | 0.32 | 0.27 | 0.40 | 0.20 | 0.27 | 0.96 |
| N3 | 0.51 | 0.63 | 0.24 | 0.31 | 0.17 | 0.33 | 0.30 | 1.25 | 0.25 | 0.26 | 0.42 | 0.21 | 0.28 | 1.00 |
| N4 | 0.44 | 0.54 | 0.22 | 0.26 | 0.15 | 0.28 | 0.26 | 1.11 | 0.22 | 0.37 | 0.45 | 0.23 | 0.30 | 0.95 |
| N5 | 0.53 | 0.54 | 0.24 | 0.33 | 0.17 | 0.21 | 0.26 | 0.89 | 0.31 | 0.31 | 0.39 | 0.24 | 0.29 | 0.84 |
| N6 | 0.76 | 0.70 | 0.30 | 0.48 | 0.23 | 0.34 | 0.31 | 1.14 | 0.38 | 0.45 | 0.47 | 0.28 | 0.40 | 1.07 |
| N7 | 0.77 | 0.72 | 0.30 | 0.45 | 0.23 | 0.25 | 0.32 | 1.14 | 0.34 | 0.28 | 0.44 | 0.21 | 0.30 | 1.04 |
| N8 | 0.72 | 0.72 | 0.29 | 0.44 | 0.22 | 0.24 | 0.39 | 1.00 | 0.35 | 0.46 | 0.33 | 0.18 | 0.36 | 1.09 |
| N9 | 0.68 | 0.64 | 0.29 | 0.42 | 0.21 | 0.23 | 0.27 | 0.91 | 0.38 | 0.39 | 0.50 | 0.17 | 0.34 | 1.01 |
| N10 | 0.71 | 0.66 | 0.29 | 0.42 | 0.20 | 0.26 | 0.28 | 1.10 | 0.36 | 0.44 | 0.30 | 0.18 | 0.34 | 1.03 |
| N11 | 0.72 | 0.68 | 0.29 | 0.44 | 0.21 | 0.26 | 0.29 | 1.11 | 0.36 | 0.28 | 0.30 | 0.30 | 0.34 | 1.04 |
| N12 | 0.70 | 0.60 | 0.29 | 0.39 | 0.22 | 0.31 | 0.28 | 1.06 | 0.39 | 0.21 | 0.30 | 0.17 | 0.32 | 0.94 |
| N13 | 0.82 | 0.65 | 0.29 | 0.38 | 0.21 | 0.28 | 0.27 | 1.08 | 0.33 | 0.36 | 0.42 | 0.19 | 0.25 | 0.97 |
| N14 | 0.76 | 0.62 | 0.31 | 0.29 | 0.24 | 0.38 | 0.29 | 1.14 | 0.38 | 0.42 | 0.47 | 0.16 | 0.34 | 1.08 |
| N15 | 0.70 | 0.57 | 0.29 | 0.33 | 0.21 | 0.30 | 0.26 | 1.00 | 0.36 | 0.45 | 0.44 | 0.15 | 0.31 | 0.93 |
| N16 | 0.56 | 0.47 | 0.25 | 0.22 | 0.19 | 0.26 | 0.23 | 0.85 | 0.25 | 0.38 | 0.46 | 0.13 | 0.31 | 0.79 |
| N17 | 0.67 | 0.57 | 0.26 | 0.29 | 0.19 | 0.25 | 0.24 | 0.96 | 0.24 | 0.31 | 0.35 | 0.18 | 0.22 | 0.89 |
| N18 | 0.75 | 0.59 | 0.30 | 0.32 | 0.22 | 0.30 | 0.36 | 1.06 | 0.34 | 0.46 | 0.58 | 0.16 | 0.31 | 1.01 |
| N19 | 0.79 | 0.61 | 0.30 | 0.27 | 0.19 | 0.31 | 0.24 | 0.93 | 0.28 | 0.41 | 0.46 | 0.21 | 0.29 | 0.90 |
| N20 | 1.00 | 0.68 | 0.33 | 0.29 | 0.19 | 0.30 | 0.24 | 1.00 | 0.34 | 0.36 | 0.39 | 0.24 | 0.27 | 0.95 |
| N21 | 1.08 | 0.63 | 0.31 | 0.28 | 0.18 | 0.31 | 0.23 | 0.92 | 0.33 | 0.45 | 0.41 | 0.21 | 0.31 | 0.91 |
| N22 | 0.99 | 0.59 | 0.30 | 0.26 | 0.17 | 0.27 | 0.22 | 0.86 | 0.31 | 0.43 | 0.39 | 0.25 | 0.30 | 0.85 |
| N23 | 1.10 | 0.62 | 0.31 | 0.27 | 0.19 | 0.29 | 0.23 | 0.99 | 0.34 | 0.45 | 0.52 | 0.26 | 0.28 | 0.98 |
| N24 | 1.03 | 0.66 | 0.30 | 0.28 | 0.20 | 0.28 | 0.24 | 0.97 | 0.28 | 0.39 | 0.42 | 0.26 | 0.31 | 0.97 |
| N25 | 1.03 | 0.64 | 0.31 | 0.28 | 0.19 | 0.29 | 0.23 | 0.93 | 0.29 | 0.40 | 0.49 | 0.26 | 0.31 | 0.93 |
| N26 | 1.14 | 0.65 | 0.31 | 0.27 | 0.19 | 0.30 | 0.24 | 1.02 | 0.33 | 0.47 | 0.54 | 0.23 | 0.31 | 1.02 |
| N27 | 1.08 | 0.61 | 0.30 | 0.26 | 0.18 | 0.28 | 0.22 | 0.96 | 0.30 | 0.41 | 0.44 | 0.26 | 0.30 | 0.96 |
| N28 | 1.01 | 0.57 | 0.29 | 0.24 | 0.17 | 0.28 | 0.21 | 0.88 | 0.29 | 0.35 | 0.37 | 0.22 | 0.16 | 0.84 |
| N29 | 1.05 | 0.60 | 0.31 | 0.26 | 0.18 | 0.28 | 0.22 | 0.93 | 0.32 | 0.44 | 0.51 | 0.30 | 0.30 | 0.93 |
| N30 | 1.14 | 0.64 | 0.32 | 0.27 | 0.19 | 0.29 | 0.23 | 1.00 | 0.33 | 0.47 | 0.54 | 0.27 | 0.32 | 1.01 |
| N31 | 1.21 | 0.67 | 0.33 | 0.27 | 0.20 | 0.31 | 0.31 | 1.05 | 0.34 | 0.46 | 0.57 | 0.33 | 0.33 | 1.06 |
| N32 | 0.91 | 0.66 | 0.31 | 0.33 | 0.22 | 0.28 | 0.26 | 0.86 | 0.34 | 0.42 | 0.43 | 0.17 | 0.14 | 1.04 |
| N33 | 0.91 | 0.69 | 0.32 | 0.35 | 0.23 | 0.30 | 0.27 | 0.90 | 0.32 | 0.44 | 0.44 | 0.18 | 0.23 | 1.09 |
| N34 | 0.97 | 0.72 | 0.33 | 0.35 | 0.23 | 0.27 | 0.27 | 0.93 | 0.31 | 0.45 | 0.46 | 0.18 | 0.24 | 1.13 |
| N35 | 0.93 | 0.71 | 0.33 | 0.36 | 0.23 | 0.30 | 0.27 | 0.91 | 0.33 | 0.45 | 0.45 | 0.18 | 0.24 | 1.09 |
| N36 | 0.88 | 0.70 | 0.32 | 0.34 | 0.22 | 0.33 | 0.26 | 0.89 | 0.27 | 0.43 | 0.43 | 0.18 | 0.23 | 1.06 |
| N37 | 0.80 | 0.69 | 0.31 | 0.32 | 0.22 | 0.32 | 0.26 | 0.88 | 0.25 | 0.40 | 0.41 | 0.17 | 0.22 | 1.03 |
| N38 | 0.81 | 0.72 | 0.32 | 0.34 | 0.23 | 0.30 | 0.27 | 0.93 | 0.27 | 0.43 | 0.44 | 0.18 | 0.23 | 1.09 |
| N39 | 0.75 | 0.56 | 0.28 | 0.29 | 0.19 | 0.22 | 0.23 | 0.71 | 0.26 | 0.38 | 0.37 | 0.15 | 0.20 | 0.85 |
| N40 | 0.55 | 0.53 | 0.20 | 0.26 | 0.13 | 0.24 | 0.23 | 0.72 | 0.27 | 0.34 | 0.32 | 0.15 | 0.21 | 0.81 |

| Label | Peak 2 | Peak 4 | Peak 7 | Peak 8 | Peak 9 | Peak 10 | Peak 11 | Peak 13 | Peak 17 | Peak 21 | Peak 23 | Peak 24 | Peak 26 | Peak 27 |
| --- | --- | --- | --- | --- | --- | --- | --- | --- | --- | --- | --- | --- | --- | --- |
| N41 | 0.57 | 0.50 | 0.19 | 0.22 | 0.14 | 0.21 | 0.21 | 0.74 | 0.26 | 0.32 | 0.35 | 0.15 | 0.21 | 0.83 |
| N42 | 0.52 | 0.51 | 0.20 | 0.20 | 0.14 | 0.23 | 0.19 | 0.75 | 0.26 | 0.31 | 0.32 | 0.14 | 0.21 | 0.84 |
| N43 | 0.72 | 0.66 | 0.25 | 0.34 | 0.20 | 0.29 | 0.27 | 0.93 | 0.37 | 0.39 | 0.41 | 0.18 | 0.26 | 1.02 |
| N44 | 0.55 | 0.52 | 0.21 | 0.28 | 0.15 | 0.25 | 0.23 | 0.92 | 0.28 | 0.32 | 0.38 | 0.15 | 0.21 | 0.84 |
| N45 | 0.76 | 0.67 | 0.28 | 0.41 | 0.21 | 0.28 | 0.27 | 0.86 | 0.34 | 0.38 | 0.38 | 0.16 | 0.24 | 0.97 |
| N46 | 0.76 | 0.64 | 0.27 | 0.40 | 0.20 | 0.27 | 0.26 | 0.84 | 0.27 | 0.38 | 0.40 | 0.17 | 0.14 | 0.94 |
| N47 | 0.74 | 0.57 | 0.26 | 0.29 | 0.20 | 0.29 | 0.22 | 0.92 | 0.24 | 0.37 | 0.39 | 0.16 | 0.22 | 1.04 |
| N48 | 0.78 | 0.59 | 0.29 | 0.32 | 0.21 | 0.25 | 0.24 | 0.92 | 0.34 | 0.40 | 0.45 | 0.18 | 0.23 | 1.08 |
| N49 | 0.74 | 0.57 | 0.27 | 0.31 | 0.20 | 0.27 | 0.23 | 0.88 | 0.27 | 0.39 | 0.43 | 0.17 | 0.21 | 1.02 |
| N50 | 0.82 | 0.65 | 0.31 | 0.36 | 0.23 | 0.33 | 0.26 | 1.02 | 0.30 | 0.42 | 0.42 | 0.19 | 0.22 | 1.16 |
| N51 | 0.87 | 0.64 | 0.31 | 0.27 | 0.20 | 0.30 | 0.23 | 0.85 | 0.27 | 0.40 | 0.42 | 0.17 | 0.21 | 1.01 |
| N52 | 1.06 | 0.76 | 0.35 | 0.32 | 0.24 | 0.33 | 0.27 | 1.03 | 0.35 | 0.47 | 0.28 | 0.19 | 0.23 | 1.21 |
| A1 | 0.49 | 0.70 | 0.33 | 0.33 | 0.21 | 0.31 | 0.25 | 0.91 | 0.25 | 0.44 | 0.44 | 0.19 | 0.25 | 1.04 |
| A2 | 0.47 | 0.59 | 0.29 | 0.28 | 0.19 | 0.31 | 0.23 | 0.76 | 0.28 | 0.39 | 0.40 | 0.18 | 0.22 | 0.88 |
| A3 | 0.56 | 0.60 | 0.30 | 0.27 | 0.19 | 0.28 | 0.23 | 0.85 | 0.37 | 0.42 | 0.44 | 0.17 | 0.14 | 0.96 |
| A4 | 0.57 | 0.63 | 0.32 | 0.28 | 0.19 | 0.28 | 0.29 | 0.85 | 0.26 | 0.45 | 0.45 | 0.18 | 0.14 | 0.99 |
| A5 | 0.52 | 0.59 | 0.30 | 0.26 | 0.18 | 0.27 | 0.22 | 0.79 | 0.25 | 0.42 | 0.38 | 0.16 | 0.21 | 0.91 |
| A6 | 0.55 | 0.61 | 0.32 | 0.28 | 0.18 | 0.26 | 0.28 | 0.80 | 0.27 | 0.44 | 0.44 | 0.17 | 0.14 | 0.94 |
| A7 | 0.40 | 0.65 | 0.33 | 0.30 | 0.20 | 0.28 | 0.29 | 0.86 | 0.28 | 0.45 | 0.47 | 0.18 | 0.23 | 1.03 |
| A8 | 0.55 | 0.63 | 0.30 | 0.33 | 0.21 | 0.25 | 0.26 | 0.82 | 0.44 | 0.45 | 0.46 | 0.19 | 0.21 | 0.99 |
| A9 | 0.58 | 0.65 | 0.31 | 0.34 | 0.22 | 0.26 | 0.26 | 0.86 | 0.44 | 0.46 | 0.48 | 0.19 | 0.21 | 1.04 |
| A10 | 0.58 | 0.69 | 0.32 | 0.35 | 0.24 | 0.27 | 0.27 | 0.90 | 0.36 | 0.48 | 0.50 | 0.19 | 0.22 | 1.09 |
| A11 | 0.58 | 0.67 | 0.33 | 0.39 | 0.22 | 0.26 | 0.27 | 0.87 | 0.45 | 0.48 | 0.49 | 0.20 | 0.16 | 1.05 |
| A12 | 0.55 | 0.64 | 0.31 | 0.34 | 0.21 | 0.25 | 0.26 | 0.85 | 0.40 | 0.44 | 0.46 | 0.18 | 0.15 | 1.00 |
| A13 | 0.50 | 0.65 | 0.30 | 0.33 | 0.22 | 0.24 | 0.26 | 0.85 | 0.29 | 0.43 | 0.45 | 0.18 | 0.24 | 1.00 |
| A14 | 0.46 | 0.55 | 0.28 | 0.30 | 0.19 | 0.23 | 0.23 | 0.70 | 0.38 | 0.41 | 0.41 | 0.17 | 0.14 | 0.85 |
| A15 | 0.34 | 0.48 | 0.19 | 0.27 | 0.14 | 0.29 | 0.22 | 0.68 | 0.39 | 0.35 | 0.35 | 0.16 | 0.14 | 0.76 |
| A16 | 0.39 | 0.53 | 0.20 | 0.28 | 0.15 | 0.22 | 0.22 | 0.79 | 0.26 | 0.35 | 0.38 | 0.16 | 0.14 | 0.87 |
| A17 | 0.38 | 0.54 | 0.21 | 0.27 | 0.15 | 0.28 | 0.26 | 0.81 | 0.27 | 0.34 | 0.38 | 0.16 | 0.14 | 0.88 |
| A18 | 0.44 | 0.65 | 0.26 | 0.38 | 0.21 | 0.27 | 0.28 | 0.92 | 0.40 | 0.44 | 0.47 | 0.21 | 0.18 | 1.01 |
| A19 | 0.38 | 0.56 | 0.23 | 0.33 | 0.16 | 0.27 | 0.24 | 1.00 | 0.24 | 0.36 | 0.42 | 0.18 | 0.19 | 0.90 |
| A20 | 0.34 | 0.67 | 0.29 | 0.46 | 0.22 | 0.26 | 0.29 | 0.88 | 0.47 | 0.44 | 0.45 | 0.21 | 0.17 | 0.98 |
| A21 | 0.48 | 0.64 | 0.28 | 0.44 | 0.22 | 0.25 | 0.27 | 0.85 | 0.44 | 0.44 | 0.44 | 0.20 | 0.17 | 0.95 |
| A22 | 0.54 | 0.61 | 0.30 | 0.37 | 0.21 | 0.32 | 0.25 | 0.96 | 0.33 | 0.43 | 0.47 | 0.18 | 0.17 | 1.10 |
| A23 | 0.54 | 0.61 | 0.30 | 0.37 | 0.21 | 0.32 | 0.24 | 0.96 | 0.34 | 0.47 | 0.47 | 0.18 | 0.17 | 1.11 |
| A24 | 0.53 | 0.67 | 0.33 | 0.42 | 0.26 | 0.30 | 0.28 | 1.03 | 0.49 | 0.51 | 0.53 | 0.22 | 0.14 | 1.23 |
| A25 | 0.65 | 0.69 | 0.34 | 0.32 | 0.22 | 0.36 | 0.36 | 0.91 | 0.36 | 0.49 | 0.50 | 0.17 | 0.16 | 1.10 |
| A26 | 0.77 | 0.83 | 0.39 | 0.37 | 0.26 | 0.38 | 0.29 | 1.10 | 0.37 | 0.57 | 0.59 | 0.22 | 0.18 | 1.32 |

**Table S4.** Pearson correlation coefficient based on RPA between 78 batches of GHIs

| Label | *r* | Label | *r* | Label | *r* |
| --- | --- | --- | --- | --- | --- |
| N1 | 0.962 | N27 | 0.949 | A1 | 0.952 |
| N2 | 0.952 | N28 | 0.941 | A2 | 0.964 |
| N3 | 0.911 | N29 | 0.943 | A3 | 0.973 |
| N4 | 0.918 | N30 | 0.949 | A4 | 0.975 |
| N5 | 0.961 | N31 | 0.943 | A5 | 0.977 |
| N6 | 0.971 | N32 | 0.983 | A6 | 0.973 |
| N7 | 0.962 | N33 | 0.991 | A7 | 0.927 |
| N8 | 0.962 | N34 | 0.988 | A8 | 0.961 |
| N9 | 0.979 | N35 | 0.989 | A9 | 0.967 |
| N10 | 0.961 | N36 | 0.990 | A10 | 0.972 |
| N11 | 0.944 | N37 | 0.992 | A11 | 0.960 |
| N12 | 0.936 | N38 | 0.994 | A12 | 0.966 |
| N13 | 0.981 | N39 | 0.985 | A13 | 0.962 |
| N14 | 0.975 | N40 | 0.988 | A14 | 0.954 |
| N15 | 0.979 | N41 | 0.992 | A15 | 0.911 |
| N16 | 0.963 | N42 | 0.985 | A16 | 0.951 |
| N17 | 0.982 | N43 | 0.989 | A17 | 0.942 |
| N18 | 0.975 | N44 | 0.974 | A18 | 0.934 |
| N19 | 0.983 | N45 | 0.986 | A19 | 0.923 |
| N20 | 0.960 | N46 | 0.986 | A20 | 0.869 |
| N21 | 0.941 | N47 | 0.997 | A21 | 0.932 |
| N22 | 0.941 | N48 | 0.998 | A22 | 0.967 |
| N23 | 0.954 | N49^*^ | 1.000 | A23 | 0.966 |
| N24 | 0.960 | N50 | 0.998 | A24 | 0.938 |
| N25 | 0.955 | N51 | 0.988 | A25 | 0.973 |
| N26 | 0.954 | N52 | 0.971 | A26 | 0.981 |

*referenced batch

**Table S5.** RLPA extracted from HPLC-ELSD fingerprints of 78 batches of GHIs

| Label | Peak 2 | Peak 3 | Peak 4 | Label | Peak 2 | Peak 3 | Peak 4 | Label | Peak 2 | Peak 3 | Peak 4 |
| --- | --- | --- | --- | --- | --- | --- | --- | --- | --- | --- | --- |
| N1 | 0.73 | 0.68 | 0.87 | N27 | 0.72 | 0.70 | 0.89 | A1 | 0.64 | 0.61 | 0.82 |
| N2 | 0.72 | 0.69 | 0.87 | N28 | 0.72 | 0.71 | 0.89 | A2 | 0.64 | 0.61 | 0.81 |
| N3 | 0.73 | 0.70 | 0.88 | N29 | 0.72 | 0.71 | 0.89 | A3 | 0.64 | 0.60 | 0.82 |
| N4 | 0.72 | 0.70 | 0.88 | N30 | 0.72 | 0.71 | 0.89 | A4 | 0.64 | 0.61 | 0.81 |
| N5 | 0.70 | 0.72 | 0.86 | N31 | 0.72 | 0.70 | 0.90 | A5 | 0.64 | 0.61 | 0.81 |
| N6 | 0.70 | 0.71 | 0.87 | N32 | 0.71 | 0.70 | 0.90 | A6 | 0.64 | 0.61 | 0.81 |
| N7 | 0.70 | 0.71 | 0.88 | N33 | 0.70 | 0.70 | 0.90 | A7 | 0.65 | 0.61 | 0.82 |
| N8 | 0.70 | 0.71 | 0.88 | N34 | 0.70 | 0.69 | 0.91 | A8 | 0.62 | 0.62 | 0.81 |
| N9 | 0.70 | 0.72 | 0.87 | N35 | 0.69 | 0.69 | 0.90 | A9 | 0.63 | 0.61 | 0.81 |
| N10 | 0.70 | 0.71 | 0.88 | N36 | 0.70 | 0.69 | 0.90 | A10 | 0.61 | 0.62 | 0.77 |
| N11 | 0.70 | 0.71 | 0.88 | N37 | 0.70 | 0.69 | 0.91 | A11 | 0.67 | 0.63 | 0.82 |
| N12 | 0.69 | 0.71 | 0.87 | N38 | 0.70 | 0.68 | 0.90 | A12 | 0.67 | 0.63 | 0.82 |
| N13 | 0.70 | 0.71 | 0.88 | N39 | 0.70 | 0.71 | 0.90 | A13 | 0.67 | 0.64 | 0.83 |
| N14 | 0.72 | 0.70 | 0.89 | N40 | 0.71 | 0.72 | 0.90 | A14 | 0.66 | 0.64 | 0.81 |
| N15 | 0.70 | 0.71 | 0.88 | N41 | 0.74 | 0.70 | 0.88 | A15 | 0.68 | 0.65 | 0.83 |
| N16 | 0.71 | 0.72 | 0.88 | N42 | 0.71 | 0.72 | 0.90 | A16 | 0.68 | 0.64 | 0.82 |
| N17 | 0.71 | 0.71 | 0.88 | N43 | 0.71 | 0.71 | 0.89 | A17 | 0.67 | 0.65 | 0.82 |
| N18 | 0.71 | 0.71 | 0.88 | N44 | 0.72 | 0.71 | 0.88 | A18 | 0.66 | 0.64 | 0.82 |
| N19 | 0.72 | 0.71 | 0.88 | N45 | 0.70 | 0.66 | 0.85 | A19 | 0.68 | 0.65 | 0.82 |
| N20 | 0.72 | 0.73 | 0.89 | N46 | 0.71 | 0.66 | 0.85 | A20 | 0.67 | 0.63 | 0.81 |
| N21 | 0.71 | 0.70 | 0.89 | N47 | 0.72 | 0.65 | 0.86 | A21 | 0.66 | 0.64 | 0.81 |
| N22 | 0.71 | 0.71 | 0.89 | N48 | 0.73 | 0.65 | 0.86 | A22 | 0.71 | 0.68 | 0.84 |
| N23 | 0.70 | 0.72 | 0.89 | N49 | 0.72 | 0.65 | 0.85 | A23 | 0.68 | 0.63 | 0.82 |
| N24 | 0.72 | 0.71 | 0.90 | N50 | 0.73 | 0.66 | 0.86 | A24 | 0.68 | 0.64 | 0.83 |
| N25 | 0.71 | 0.71 | 0.89 | N51 | 0.73 | 0.65 | 0.88 | A25 | 0.68 | 0.63 | 0.84 |
| N26 | 0.72 | 0.71 | 0.89 | N52 | 0.75 | 0.68 | 0.90 | A26 | 0.68 | 0.62 | 0.83 |

*referenced batch

**Table S6.** Pearson correlation coefficient based on RLPA between 78 batches of GHIs

| Label | *r* | Label | *r* | Label | *r* |
| --- | --- | --- | --- | --- | --- |
| N1 | 0.997 | N27 | 0.969 | A1 | 0.979 |
| N2 | 0.988 | N28 | 0.960 | A2 | 0.976 |
| N3 | 0.972 | N29 | 0.953 | A3 | 0.984 |
| N4 | 0.961 | N30 | 0.949 | A4 | 0.981 |
| N5 | 0.911 | N31 | 0.968 | A5 | 0.982 |
| N6 | 0.914 | N32 | 0.946 | A6 | 0.977 |
| N7 | 0.910 | N33 | 0.946 | A7 | 0.989 |
| N8 | 0.908 | N34 | 0.953 | A8 | 0.950 |
| N9 | 0.895 | N35 | 0.939 | A9 | 0.970 |
| N10 | 0.911 | N36 | 0.942 | A10 | 0.936 |
| N11 | 0.913 | N37 | 0.951 | A11 | 0.989 |
| N12 | 0.896 | N38 | 0.957 | A12 | 0.988 |
| N13 | 0.917 | N39 | 0.909 | A13 | 0.986 |
| N14 | 0.956 | N40 | 0.928 | A14 | 0.972 |
| N15 | 0.924 | N41 | 0.989 | A15 | 0.979 |
| N16 | 0.918 | N42 | 0.922 | A16 | 0.991 |
| N17 | 0.953 | N43 | 0.927 | A17 | 0.969 |
| N18 | 0.945 | N44 | 0.956 | A18 | 0.969 |
| N19 | 0.947 | N45 | 0.990 | A19 | 0.985 |
| N20 | 0.919 | N46 | 0.995 | A20 | 0.983 |
| N21 | 0.955 | N47 | 1.000 | A21 | 0.977 |
| N22 | 0.938 | N48 | 0.998 | A22 | 0.986 |
| N23 | 0.903 | N49^*^ | 1.000 | A23 | 0.993 |
| N24 | 0.954 | N50 | 0.999 | A24 | 0.991 |
| N25 | 0.946 | N51 | 1.000 | A25 | 0.993 |
| N26 | 0.951 | N52 | 0.999 | A26 | 0.998 |

*referenced batch

**Table S7.** Absolute concentrations of compounds from 78 batches of GHIs (mg·mL^-1^)

| Label | Adenosine | Hydroxysafflor yellow A | α-Glucose | β-Glucose | Aceglutamide | Alanine | 1,2-Propanediol | Valine |
| --- | --- | --- | --- | --- | --- | --- | --- | --- |
| N1 | 0.0131 | 0.0723 | 0.2708 | 0.3550 | 10.6583 | 0.0302 | 4.5972 | 0.0193 |
| N2 | 0.0140 | 0.0561 | 0.2829 | 0.3149 | 9.9845 | 0.0285 | 4.6020 | 0.0312 |
| N3 | 0.0086 | 0.0720 | 0.2625 | 0.3332 | 10.0728 | 0.0216 | 4.4730 | 0.0243 |
| N4 | 0.0102 | 0.0569 | 0.2397 | 0.3036 | 10.0874 | 0.0224 | 4.3627 | 0.0225 |
| N5 | 0.0105 | 0.0748 | 0.2164 | 0.2804 | 9.3257 | 0.0286 | 4.8053 | 0.0194 |
| N6 | 0.0118 | 0.0507 | 0.2021 | 0.2497 | 9.2418 | 0.0264 | 4.4207 | 0.0280 |
| N7 | 0.0130 | 0.0512 | 0.2146 | 0.2572 | 9.1037 | 0.0271 | 4.4349 | 0.0235 |
| N8 | 0.0103 | 0.0538 | 0.2232 | 0.2520 | 9.2603 | 0.0270 | 4.3911 | 0.0235 |
| N9 | 0.0087 | 0.0481 | 0.2068 | 0.2392 | 8.9532 | 0.0251 | 4.2630 | 0.0204 |
| N10 | 0.0108 | 0.0588 | 0.2373 | 0.2619 | 9.5460 | 0.0272 | 4.8093 | 0.0214 |
| N11 | 0.0108 | 0.0517 | 0.2129 | 0.2498 | 9.0579 | 0.0260 | 4.3977 | 0.0218 |
| N12 | 0.0106 | 0.0548 | 0.2154 | 0.2442 | 9.2853 | 0.0259 | 4.4426 | 0.0202 |
| N13 | 0.0125 | 0.0585 | 0.2280 | 0.2682 | 10.3996 | 0.0274 | 4.4420 | 0.0204 |
| N14 | 0.0119 | 0.0548 | 0.2545 | 0.2869 | 9.3135 | 0.0275 | 4.4723 | 0.0237 |
| N15 | 0.0096 | 0.0533 | 0.2428 | 0.2607 | 9.6105 | 0.0267 | 4.4017 | 0.0213 |
| N16 | 0.0094 | 0.0600 | 0.2296 | 0.3076 | 9.1029 | 0.0263 | 4.6707 | 0.0279 |
| N17 | 0.0111 | 0.0574 | 0.2574 | 0.2829 | 8.9869 | 0.0272 | 4.3625 | 0.0240 |
| N18 | 0.0108 | 0.0676 | 0.2388 | 0.2832 | 10.1444 | 0.0262 | 4.4942 | 0.0312 |
| N19 | 0.0118 | 0.0577 | 0.2473 | 0.2965 | 9.5327 | 0.0274 | 4.3083 | 0.0240 |
| N20 | 0.0131 | 0.0700 | 0.2646 | 0.2758 | 10.3106 | 0.0319 | 4.8321 | 0.0277 |
| N21 | 0.0130 | 0.0676 | 0.2289 | 0.2561 | 9.9296 | 0.0321 | 4.4221 | 0.0213 |
| N22 | 0.0105 | 0.0673 | 0.2178 | 0.2376 | 9.8017 | 0.0299 | 4.2847 | 0.0263 |
| N23 | 0.0121 | 0.0525 | 0.2270 | 0.2497 | 9.0301 | 0.0322 | 4.6807 | 0.0247 |

| Label | Adenosine | Hydroxysafflor yellow A | α-Glucose | β-Glucose | Aceglutamide | L-Alanine | 1,2-Propanediol | L-Valine |
| --- | --- | --- | --- | --- | --- | --- | --- | --- |
| N24 | 0.0121 | 0.0504 | 0.2169 | 0.2247 | 9.7187 | 0.0235 | 4.5214 | 0.0217 |
| N25 | 0.0123 | 0.0587 | 0.2327 | 0.2591 | 9.0637 | 0.0299 | 4.3122 | 0.0297 |
| N26 | 0.0106 | 0.0629 | 0.2378 | 0.2675 | 9.9687 | 0.0306 | 4.6799 | 0.0206 |
| N27 | 0.0147 | 0.0553 | 0.2524 | 0.2578 | 9.7716 | 0.0289 | 4.6202 | 0.0256 |
| N28 | 0.0140 | 0.0689 | 0.2492 | 0.2572 | 9.5167 | 0.0290 | 4.7368 | 0.0251 |
| N29 | 0.0121 | 0.0603 | 0.2367 | 0.2522 | 9.8648 | 0.0273 | 4.4867 | 0.0291 |
| N30 | 0.0136 | 0.0626 | 0.2500 | 0.2534 | 9.6715 | 0.0294 | 4.4907 | 0.0243 |
| N31 | 0.0139 | 0.0791 | 0.2775 | 0.2524 | 9.7310 | 0.0297 | 4.5463 | 0.0294 |
| N32 | 0.0139 | 0.0517 | 0.2465 | 0.2559 | 9.4124 | 0.0285 | 4.3085 | 0.0248 |
| N33 | 0.0133 | 0.0639 | 0.2310 | 0.2395 | 9.3757 | 0.0295 | 4.6108 | 0.0234 |
| N34 | 0.0124 | 0.0543 | 0.2178 | 0.2330 | 9.8112 | 0.0280 | 4.3600 | 0.0239 |
| N35 | 0.0132 | 0.0788 | 0.2018 | 0.2371 | 8.7042 | 0.0282 | 4.1748 | 0.0179 |
| N36 | 0.0110 | 0.0621 | 0.2152 | 0.2498 | 10.0181 | 0.0290 | 4.5052 | 0.0371 |
| N37 | 0.0132 | 0.0481 | 0.1951 | 0.2147 | 7.7004 | 0.0251 | 3.8655 | 0.0248 |
| N38 | 0.0096 | 0.0421 | 0.1999 | 0.2128 | 8.0776 | 0.0245 | 3.6674 | 0.0263 |
| N39 | 0.0112 | 0.0439 | 0.2021 | 0.2222 | 8.1949 | 0.0246 | 3.4942 | 0.0232 |
| N40 | 0.0090 | 0.0468 | 0.1756 | 0.1891 | 7.6511 | 0.0230 | 3.5740 | 0.0247 |
| N41 | 0.0092 | 0.0414 | 0.1782 | 0.1824 | 7.8149 | 0.0241 | 3.7042 | 0.0196 |
| N42 | 0.0087 | 0.0434 | 0.2255 | 0.2566 | 7.9119 | 0.0250 | 3.6067 | 0.0155 |
| N43 | 0.0113 | 0.0458 | 0.2391 | 0.2569 | 8.0236 | 0.0259 | 2.9904 | 0.0151 |
| N44 | 0.0103 | 0.0546 | 0.2104 | 0.2246 | 7.7393 | 0.0233 | 3.8164 | 0.0234 |
| N45 | 0.0096 | 0.0427 | 0.2468 | 0.2902 | 7.9533 | 0.0208 | 3.9882 | 0.0220 |
| N46 | 0.0101 | 0.0327 | 0.2281 | 0.2482 | 7.5798 | 0.0246 | 3.6188 | 0.0190 |
| N47 | 0.0090 | 0.0374 | 0.1870 | 0.2373 | 8.4565 | 0.0246 | 3.7802 | 0.0203 |

| Label | Adenosine | Hydroxysafflor yellow A | α-Glucose | β-Glucose | Aceglutamide | Alanine | 1,2-Propanediol | Valine |
| --- | --- | --- | --- | --- | --- | --- | --- | --- |
| N48 | 0.0091 | 0.0406 | 0.1897 | 0.2110 | 7.7686 | 0.0225 | 3.7803 | 0.0167 |
| N49 | 0.0088 | 0.0434 | 0.2063 | 0.2259 | 7.9848 | 0.0204 | 3.7304 | 0.0200 |
| N50 | 0.0095 | 0.0406 | 0.2194 | 0.2513 | 8.5424 | 0.0226 | 3.8192 | 0.0269 |
| N51 | 0.0135 | 0.0587 | 0.2085 | 0.2228 | 7.7622 | 0.0249 | 3.6305 | 0.0214 |
| N52 | 0.0108 | 0.0341 | 0.1969 | 0.2110 | 7.8747 | 0.0240 | 3.9242 | 0.0185 |
| A1 | 0.0096 | 0.0330 | 0.2887 | 0.3568 | 8.0725 | 0.0331 | 4.6301 | 0.0171 |
| A2 | 0.0089 | 0.0712 | 0.3054 | 0.3275 | 7.6865 | 0.0370 | 4.6563 | 0.0167 |
| A3 | 0.0053 | 0.0744 | 0.3051 | 0.3486 | 7.0587 | 0.0362 | 4.6072 | 0.0148 |
| A4 | 0.0067 | 0.0892 | 0.2927 | 0.3237 | 7.0066 | 0.0337 | 4.5060 | 0.0139 |
| A5 | 0.0111 | 0.0787 | 0.2824 | 0.3161 | 7.0553 | 0.0324 | 4.6125 | 0.0158 |
| A6 | 0.0087 | 0.0739 | 0.3016 | 0.3457 | 7.0599 | 0.0367 | 4.8568 | 0.0157 |
| A7 | 0.0067 | 0.0767 | 0.2634 | 0.2850 | 8.1269 | 0.0312 | 3.8310 | 0.0137 |
| A8 | 0.0062 | 0.0670 | 0.2626 | 0.2656 | 7.7183 | 0.0324 | 5.0860 | 0.0144 |
| A9 | 0.0083 | 0.0702 | 0.2814 | 0.2983 | 6.9757 | 0.0327 | 4.5997 | 0.0150 |
| A10 | 0.0079 | 0.0759 | 0.2470 | 0.2888 | 6.9843 | 0.0325 | 4.3608 | 0.0167 |
| A11 | 0.0055 | 0.0587 | 0.2726 | 0.3085 | 6.6642 | 0.0337 | 4.5515 | 0.0175 |
| A12 | 0.0035 | 0.0572 | 0.2855 | 0.3356 | 7.5934 | 0.0354 | 5.1787 | 0.0161 |
| A13 | 0.0093 | 0.1029 | 0.3227 | 0.4047 | 6.7750 | 0.0355 | 4.6838 | 0.0126 |
| A14 | 0.0097 | 0.0809 | 0.2651 | 0.2989 | 7.3538 | 0.0340 | 4.4614 | 0.0171 |
| A15 | 0.0067 | 0.0778 | 0.3199 | 0.4053 | 7.3858 | 0.0365 | 4.7037 | 0.0126 |
| A16 | 0.0087 | 0.1043 | 0.3248 | 0.3418 | 8.1874 | 0.0347 | 4.9734 | 0.0127 |
| A17 | 0.0055 | 0.0679 | 0.2789 | 0.3476 | 8.0936 | 0.0318 | 4.8041 | 0.0144 |
| A18 | 0.0084 | 0.0450 | 0.3305 | 0.3692 | 7.3997 | 0.0341 | 4.8905 | 0.0171 |
| A19 | 0.0089 | 0.0648 | 0.3683 | 0.3966 | 8.0740 | 0.0290 | 4.9505 | 0.0150 |

| Label | Adenosine | Hydroxysafflor yellow A | α-Glucose | β-Glucose | Aceglutamide | Alanine | 1,2-Propanediol | Valine |
| --- | --- | --- | --- | --- | --- | --- | --- | --- |
| A20 | 0.0105 | 0.0873 | 0.2685 | 0.3171 | 8.5554 | 0.0350 | 4.6376 | 0.0132 |
| A21 | 0.0055 | 0.0648 | 0.2584 | 0.3022 | 7.4950 | 0.0305 | 4.3147 | 0.0173 |
| A22 | 0.0074 | 0.0772 | 0.3229 | 0.3578 | 7.5941 | 0.0311 | 4.8078 | 0.0149 |
| A23 | 0.0079 | 0.0774 | 0.2997 | 0.3254 | 7.6725 | 0.0297 | 4.5900 | 0.0140 |
| A24 | 0.0065 | 0.0606 | 0.3073 | 0.3320 | 7.8777 | 0.0318 | 4.4689 | 0.0116 |
| A25 | 0.0098 | 0.0821 | 0.2564 | 0.3320 | 7.5026 | 0.0326 | 4.9512 | 0.0172 |
| A26 | 0.0069 | 0.0813 | 0.3179 | 0.3614 | 7.2860 | 0.0364 | 4.4579 | 0.0173 |

**Table S8.** Pearson correlation coefficient based on content between 78 batches of GHIs

| Label | *r* | Label | *r* | Label | *r* |
| --- | --- | --- | --- | --- | --- |
| N1 | 0.9994 | N27 | 1.0000 | A1 | 0.9955 |
| N2 | 1.0000 | N28 | 0.9996 | A2 | 0.9926 |
| N3 | 0.9997 | N29 | 0.9999 | A3 | 0.9872 |
| N4 | 0.9995 | N30 | 1.0000 | A4 | 0.9884 |
| N5 | 0.9990 | N31 | 1.0000 | A5 | 0.9871 |
| N6 | 0.9999 | N32 | 1.0000 | A6 | 0.9823 |
| N7 | 0.9998 | N33 | 0.9997 | A7 | 1.0000 |
| N8 | 1.0000 | N34 | 0.9998 | A8 | 0.9863 |
| N9 | 1.0000 | N35 | 0.9999 | A9 | 0.9863 |
| N10 | 0.9994 | N36 | 0.9999 | A10 | 0.9906 |
| N11 | 0.9999 | N37 | 0.9995 | A11 | 0.9830 |
| N12 | 0.9999 | N38 | 0.9999 | A12 | 0.9832 |
| N13 | 0.9993 | N39 | 0.9993 | A13 | 0.9818 |
| N14 | 0.9999 | N40 | 1.0000 | A14 | 0.9926 |
| N15 | 1.0000 | N41 | 1.0000 | A15 | 0.9891 |
| N16 | 0.9991 | N42 | 0.9999 | A16 | 0.9925 |
| N17 | 0.9999 | N43 | 0.9959 | A17 | 0.9938 |
| N18 | 0.9997 | N44 | 0.9997 | A18 | 0.9861 |
| N19 | 0.9999 | N45 | 0.9995 | A19 | 0.9918 |
| N20 | 1.0000 | N46 | 0.9999 | A20 | 0.9978 |
| N21 | 0.9998 | N47 | 0.9998 | A21 | 0.9954 |
| N22 | 0.9988 | N48 | 0.9998 | A22 | 0.9896 |
| N23 | 0.9989 | N49^*^ | 1.0000 | A23 | 0.9934 |
| N24 | 1.0000 | N50 | 0.9998 | A24 | 0.9960 |
| N25 | 1.0000 | N51 | 1.0000 | A25 | 0.9862 |
| N26 | 1.0000 | N52 | 0.9996 | A26 | 0.9920 |

*referenced batch

**Table S9.** Fused feature table

| Label | HPLC-UV | | | | | | | | | | | | | | HPLC-ELSD | | | q^1^HNMR | | | | | | | |
| --- | --- | --- | --- | --- | --- | --- | --- | --- | --- | --- | --- | --- | --- | --- | --- | --- | --- | --- | --- | --- | --- | --- | --- | --- | --- |
|  | F1 | F2 | F3 | F4 | F5 | F6 | F7 | F8 | F9 | F10 | F11 | F12 | F13 | F14 | F15 | F16 | F17 | F18 | F19 | F20 | F21 | F22 | F23 | F24 | F25 |
| N1 | 0.78 | 0.48 | 0.19 | 0.19 | 0.13 | 0.22 | 0.21 | 0.84 | 0.27 | 0.30 | 0.33 | 0.16 | 0.22 | 0.75 | 0.73 | 0.68 | 0.87 | 0.0131 | 0.0723 | 0.2708 | 0.3550 | 10.6583 | 0.0302 | 4.5972 | 0.0193 |
| N2 | 0.58 | 0.63 | 0.24 | 0.29 | 0.18 | 0.33 | 0.28 | 1.07 | 0.32 | 0.27 | 0.40 | 0.20 | 0.27 | 0.96 | 0.72 | 0.69 | 0.87 | 0.0140 | 0.0561 | 0.2829 | 0.3149 | 9.9845 | 0.0285 | 4.6020 | 0.0312 |
| N3 | 0.51 | 0.63 | 0.24 | 0.31 | 0.17 | 0.33 | 0.30 | 1.25 | 0.25 | 0.26 | 0.42 | 0.21 | 0.28 | 1.00 | 0.73 | 0.70 | 0.88 | 0.0086 | 0.0720 | 0.2625 | 0.3332 | 10.0728 | 0.0216 | 4.4730 | 0.0243 |
| N4 | 0.44 | 0.54 | 0.22 | 0.26 | 0.15 | 0.28 | 0.26 | 1.11 | 0.22 | 0.37 | 0.45 | 0.23 | 0.30 | 0.95 | 0.72 | 0.70 | 0.88 | 0.0102 | 0.0569 | 0.2397 | 0.3036 | 10.0874 | 0.0224 | 4.3627 | 0.0225 |
| N5 | 0.53 | 0.54 | 0.24 | 0.33 | 0.17 | 0.21 | 0.26 | 0.89 | 0.31 | 0.31 | 0.39 | 0.24 | 0.29 | 0.84 | 0.70 | 0.72 | 0.86 | 0.0105 | 0.0748 | 0.2164 | 0.2804 | 9.3257 | 0.0286 | 4.8053 | 0.0194 |
| N6 | 0.76 | 0.70 | 0.30 | 0.48 | 0.23 | 0.34 | 0.31 | 1.14 | 0.38 | 0.45 | 0.47 | 0.28 | 0.40 | 1.07 | 0.70 | 0.71 | 0.87 | 0.0118 | 0.0507 | 0.2021 | 0.2497 | 9.2418 | 0.0264 | 4.4207 | 0.0280 |
| N7 | 0.77 | 0.72 | 0.30 | 0.45 | 0.23 | 0.25 | 0.32 | 1.14 | 0.34 | 0.28 | 0.44 | 0.21 | 0.30 | 1.04 | 0.70 | 0.71 | 0.88 | 0.0130 | 0.0512 | 0.2146 | 0.2572 | 9.1037 | 0.0271 | 4.4349 | 0.0235 |
| N8 | 0.72 | 0.72 | 0.29 | 0.44 | 0.22 | 0.24 | 0.39 | 1.00 | 0.35 | 0.46 | 0.33 | 0.18 | 0.36 | 1.09 | 0.70 | 0.71 | 0.88 | 0.0103 | 0.0538 | 0.2232 | 0.2520 | 9.2603 | 0.0270 | 4.3911 | 0.0235 |
| N9 | 0.68 | 0.64 | 0.29 | 0.42 | 0.21 | 0.23 | 0.27 | 0.91 | 0.38 | 0.39 | 0.50 | 0.17 | 0.34 | 1.01 | 0.70 | 0.72 | 0.87 | 0.0087 | 0.0481 | 0.2068 | 0.2392 | 8.9532 | 0.0251 | 4.2630 | 0.0204 |
| N10 | 0.71 | 0.66 | 0.29 | 0.42 | 0.20 | 0.26 | 0.28 | 1.10 | 0.36 | 0.44 | 0.30 | 0.18 | 0.34 | 1.03 | 0.70 | 0.71 | 0.88 | 0.0108 | 0.0588 | 0.2373 | 0.2619 | 9.5460 | 0.0272 | 4.8093 | 0.0214 |
| N11 | 0.72 | 0.68 | 0.29 | 0.44 | 0.21 | 0.26 | 0.29 | 1.11 | 0.36 | 0.28 | 0.30 | 0.30 | 0.34 | 1.04 | 0.70 | 0.71 | 0.88 | 0.0108 | 0.0517 | 0.2129 | 0.2498 | 9.0579 | 0.0260 | 4.3977 | 0.0218 |
| N12 | 0.70 | 0.60 | 0.29 | 0.39 | 0.22 | 0.31 | 0.28 | 1.06 | 0.39 | 0.21 | 0.30 | 0.17 | 0.32 | 0.94 | 0.69 | 0.71 | 0.87 | 0.0106 | 0.0548 | 0.2154 | 0.2442 | 9.2853 | 0.0259 | 4.4426 | 0.0202 |
| N13 | 0.82 | 0.65 | 0.29 | 0.38 | 0.21 | 0.28 | 0.27 | 1.08 | 0.33 | 0.36 | 0.42 | 0.19 | 0.25 | 0.97 | 0.70 | 0.71 | 0.88 | 0.0125 | 0.0585 | 0.2280 | 0.2682 | 10.3996 | 0.0274 | 4.4420 | 0.0204 |
| N14 | 0.76 | 0.62 | 0.31 | 0.29 | 0.24 | 0.38 | 0.29 | 1.14 | 0.38 | 0.42 | 0.47 | 0.16 | 0.34 | 1.08 | 0.72 | 0.70 | 0.89 | 0.0119 | 0.0548 | 0.2545 | 0.2869 | 9.3135 | 0.0275 | 4.4723 | 0.0237 |
| N15 | 0.70 | 0.57 | 0.29 | 0.33 | 0.21 | 0.30 | 0.26 | 1.00 | 0.36 | 0.45 | 0.44 | 0.15 | 0.31 | 0.93 | 0.70 | 0.71 | 0.88 | 0.0096 | 0.0533 | 0.2428 | 0.2607 | 9.6105 | 0.0267 | 4.4017 | 0.0213 |
| N16 | 0.56 | 0.47 | 0.25 | 0.22 | 0.19 | 0.26 | 0.23 | 0.85 | 0.25 | 0.38 | 0.46 | 0.13 | 0.31 | 0.79 | 0.71 | 0.72 | 0.88 | 0.0094 | 0.0600 | 0.2296 | 0.3076 | 9.1029 | 0.0263 | 4.6707 | 0.0279 |
| N17 | 0.67 | 0.57 | 0.26 | 0.29 | 0.19 | 0.25 | 0.24 | 0.96 | 0.24 | 0.31 | 0.35 | 0.18 | 0.22 | 0.89 | 0.71 | 0.71 | 0.88 | 0.0111 | 0.0574 | 0.2574 | 0.2829 | 8.9869 | 0.0272 | 4.3625 | 0.0240 |
| N18 | 0.75 | 0.59 | 0.30 | 0.32 | 0.22 | 0.30 | 0.36 | 1.06 | 0.34 | 0.46 | 0.58 | 0.16 | 0.31 | 1.01 | 0.71 | 0.71 | 0.88 | 0.0108 | 0.0676 | 0.2388 | 0.2832 | 10.1444 | 0.0262 | 4.4942 | 0.0312 |
| N19 | 0.79 | 0.61 | 0.30 | 0.27 | 0.19 | 0.31 | 0.24 | 0.93 | 0.28 | 0.41 | 0.46 | 0.21 | 0.29 | 0.90 | 0.72 | 0.71 | 0.88 | 0.0118 | 0.0577 | 0.2473 | 0.2965 | 9.5327 | 0.0274 | 4.3083 | 0.0240 |
| N20 | 1.00 | 0.68 | 0.33 | 0.29 | 0.19 | 0.30 | 0.24 | 1.00 | 0.34 | 0.36 | 0.39 | 0.24 | 0.27 | 0.95 | 0.72 | 0.73 | 0.89 | 0.0131 | 0.0700 | 0.2646 | 0.2758 | 10.3106 | 0.0319 | 4.8321 | 0.0277 |
| N21 | 1.08 | 0.63 | 0.31 | 0.28 | 0.18 | 0.31 | 0.23 | 0.92 | 0.33 | 0.45 | 0.41 | 0.21 | 0.31 | 0.91 | 0.71 | 0.70 | 0.89 | 0.0130 | 0.0676 | 0.2289 | 0.2561 | 9.9296 | 0.0321 | 4.4221 | 0.0213 |
| N22 | 0.99 | 0.59 | 0.30 | 0.26 | 0.17 | 0.27 | 0.22 | 0.86 | 0.31 | 0.43 | 0.39 | 0.25 | 0.30 | 0.85 | 0.71 | 0.71 | 0.89 | 0.0105 | 0.0673 | 0.2178 | 0.2376 | 9.8017 | 0.0299 | 4.2847 | 0.0263 |
| N23 | 1.10 | 0.62 | 0.31 | 0.27 | 0.19 | 0.29 | 0.23 | 0.99 | 0.34 | 0.45 | 0.52 | 0.26 | 0.28 | 0.98 | 0.70 | 0.72 | 0.89 | 0.0121 | 0.0525 | 0.2270 | 0.2497 | 9.0301 | 0.0322 | 4.6807 | 0.0247 |
| Label | HPLC-UV | | | | | | | | | | | | | | HPLC-ELSD | | | q^1^HNMR | | | | | | | |
|  | F1 | F2 | F3 | F4 | F5 | F6 | F7 | F8 | F9 | F10 | F11 | F12 | F13 | F14 | F15 | F16 | F17 | F18 | F19 | F20 | F21 | F22 | F23 | F24 | F25 |
| N24 | 1.03 | 0.66 | 0.30 | 0.28 | 0.20 | 0.28 | 0.24 | 0.97 | 0.28 | 0.39 | 0.42 | 0.26 | 0.31 | 0.97 | 0.72 | 0.71 | 0.90 | 0.0121 | 0.0504 | 0.2169 | 0.2247 | 9.7187 | 0.0235 | 4.5214 | 0.0217 |
| N25 | 1.03 | 0.64 | 0.31 | 0.28 | 0.19 | 0.29 | 0.23 | 0.93 | 0.29 | 0.40 | 0.49 | 0.26 | 0.31 | 0.93 | 0.71 | 0.71 | 0.89 | 0.0123 | 0.0587 | 0.2327 | 0.2591 | 9.0637 | 0.0299 | 4.3122 | 0.0297 |
| N26 | 1.14 | 0.65 | 0.31 | 0.27 | 0.19 | 0.30 | 0.24 | 1.02 | 0.33 | 0.47 | 0.54 | 0.23 | 0.31 | 1.02 | 0.72 | 0.71 | 0.89 | 0.0106 | 0.0629 | 0.2378 | 0.2675 | 9.9687 | 0.0306 | 4.6799 | 0.0206 |
| N27 | 1.08 | 0.61 | 0.30 | 0.26 | 0.18 | 0.28 | 0.22 | 0.96 | 0.30 | 0.41 | 0.44 | 0.26 | 0.30 | 0.96 | 0.72 | 0.70 | 0.89 | 0.0147 | 0.0553 | 0.2524 | 0.2578 | 9.7716 | 0.0289 | 4.6202 | 0.0256 |
| N28 | 1.01 | 0.57 | 0.29 | 0.24 | 0.17 | 0.28 | 0.21 | 0.88 | 0.29 | 0.35 | 0.37 | 0.22 | 0.16 | 0.84 | 0.72 | 0.71 | 0.89 | 0.0140 | 0.0689 | 0.2492 | 0.2572 | 9.5167 | 0.0290 | 4.7368 | 0.0251 |
| N29 | 1.05 | 0.60 | 0.31 | 0.26 | 0.18 | 0.28 | 0.22 | 0.93 | 0.32 | 0.44 | 0.51 | 0.30 | 0.30 | 0.93 | 0.72 | 0.71 | 0.89 | 0.0121 | 0.0603 | 0.2367 | 0.2522 | 9.8648 | 0.0273 | 4.4867 | 0.0291 |
| N30 | 1.14 | 0.64 | 0.32 | 0.27 | 0.19 | 0.29 | 0.23 | 1.00 | 0.33 | 0.47 | 0.54 | 0.27 | 0.32 | 1.01 | 0.72 | 0.71 | 0.89 | 0.0136 | 0.0626 | 0.2500 | 0.2534 | 9.6715 | 0.0294 | 4.4907 | 0.0243 |
| N31 | 1.21 | 0.67 | 0.33 | 0.27 | 0.20 | 0.31 | 0.31 | 1.05 | 0.34 | 0.46 | 0.57 | 0.33 | 0.33 | 1.06 | 0.72 | 0.70 | 0.90 | 0.0139 | 0.0791 | 0.2775 | 0.2524 | 9.7310 | 0.0297 | 4.5463 | 0.0294 |
| N32 | 0.91 | 0.66 | 0.31 | 0.33 | 0.22 | 0.28 | 0.26 | 0.86 | 0.34 | 0.42 | 0.43 | 0.17 | 0.14 | 1.04 | 0.71 | 0.70 | 0.90 | 0.0139 | 0.0517 | 0.2465 | 0.2559 | 9.4124 | 0.0285 | 4.3085 | 0.0248 |
| N33 | 0.91 | 0.69 | 0.32 | 0.35 | 0.23 | 0.30 | 0.27 | 0.90 | 0.32 | 0.44 | 0.44 | 0.18 | 0.23 | 1.09 | 0.70 | 0.70 | 0.90 | 0.0133 | 0.0639 | 0.2310 | 0.2395 | 9.3757 | 0.0295 | 4.6108 | 0.0234 |
| N34 | 0.97 | 0.72 | 0.33 | 0.35 | 0.23 | 0.27 | 0.27 | 0.93 | 0.31 | 0.45 | 0.46 | 0.18 | 0.24 | 1.13 | 0.70 | 0.69 | 0.91 | 0.0124 | 0.0543 | 0.2178 | 0.2330 | 9.8112 | 0.0280 | 4.3600 | 0.0239 |
| N35 | 0.93 | 0.71 | 0.33 | 0.36 | 0.23 | 0.30 | 0.27 | 0.91 | 0.33 | 0.45 | 0.45 | 0.18 | 0.24 | 1.09 | 0.69 | 0.69 | 0.90 | 0.0132 | 0.0788 | 0.2018 | 0.2371 | 8.7042 | 0.0282 | 4.1748 | 0.0179 |
| N36 | 0.88 | 0.70 | 0.32 | 0.34 | 0.22 | 0.33 | 0.26 | 0.89 | 0.27 | 0.43 | 0.43 | 0.18 | 0.23 | 1.06 | 0.70 | 0.69 | 0.90 | 0.0110 | 0.0621 | 0.2152 | 0.2498 | 10.0181 | 0.0290 | 4.5052 | 0.0371 |
| N37 | 0.80 | 0.69 | 0.31 | 0.32 | 0.22 | 0.32 | 0.26 | 0.88 | 0.25 | 0.40 | 0.41 | 0.17 | 0.22 | 1.03 | 0.70 | 0.69 | 0.91 | 0.0132 | 0.0481 | 0.1951 | 0.2147 | 7.7004 | 0.0251 | 3.8655 | 0.0248 |
| N38 | 0.81 | 0.72 | 0.32 | 0.34 | 0.23 | 0.30 | 0.27 | 0.93 | 0.27 | 0.43 | 0.44 | 0.18 | 0.23 | 1.09 | 0.70 | 0.68 | 0.90 | 0.0096 | 0.0421 | 0.1999 | 0.2128 | 8.0776 | 0.0245 | 3.6674 | 0.0263 |
| N39 | 0.75 | 0.56 | 0.28 | 0.29 | 0.19 | 0.22 | 0.23 | 0.71 | 0.26 | 0.38 | 0.37 | 0.15 | 0.20 | 0.85 | 0.70 | 0.71 | 0.90 | 0.0112 | 0.0439 | 0.2021 | 0.2222 | 8.1949 | 0.0246 | 3.4942 | 0.0232 |
| N40 | 0.55 | 0.53 | 0.20 | 0.26 | 0.13 | 0.24 | 0.23 | 0.72 | 0.27 | 0.34 | 0.32 | 0.15 | 0.21 | 0.81 | 0.71 | 0.72 | 0.90 | 0.0090 | 0.0468 | 0.1756 | 0.1891 | 7.6511 | 0.0230 | 3.5740 | 0.0247 |
| N41 | 0.57 | 0.50 | 0.19 | 0.22 | 0.14 | 0.21 | 0.21 | 0.74 | 0.26 | 0.32 | 0.35 | 0.15 | 0.21 | 0.83 | 0.74 | 0.70 | 0.88 | 0.0092 | 0.0414 | 0.1782 | 0.1824 | 7.8149 | 0.0241 | 3.7042 | 0.0196 |
| N42 | 0.52 | 0.51 | 0.20 | 0.20 | 0.14 | 0.23 | 0.19 | 0.75 | 0.26 | 0.31 | 0.32 | 0.14 | 0.21 | 0.84 | 0.71 | 0.72 | 0.90 | 0.0087 | 0.0434 | 0.2255 | 0.2566 | 7.9119 | 0.0250 | 3.6067 | 0.0155 |
| N43 | 0.72 | 0.66 | 0.25 | 0.34 | 0.20 | 0.29 | 0.27 | 0.93 | 0.37 | 0.39 | 0.41 | 0.18 | 0.26 | 1.02 | 0.71 | 0.71 | 0.89 | 0.0113 | 0.0458 | 0.2391 | 0.2569 | 8.0236 | 0.0259 | 2.9904 | 0.0151 |
| N44 | 0.55 | 0.52 | 0.21 | 0.28 | 0.15 | 0.25 | 0.23 | 0.92 | 0.28 | 0.32 | 0.38 | 0.15 | 0.21 | 0.84 | 0.72 | 0.71 | 0.88 | 0.0103 | 0.0546 | 0.2104 | 0.2246 | 7.7393 | 0.0233 | 3.8164 | 0.0234 |
| N45 | 0.76 | 0.67 | 0.28 | 0.41 | 0.21 | 0.28 | 0.27 | 0.86 | 0.34 | 0.38 | 0.38 | 0.16 | 0.24 | 0.97 | 0.70 | 0.66 | 0.85 | 0.0096 | 0.0427 | 0.2468 | 0.2902 | 7.9533 | 0.0208 | 3.9882 | 0.0220 |
| N46 | 0.76 | 0.64 | 0.27 | 0.40 | 0.20 | 0.27 | 0.26 | 0.84 | 0.27 | 0.38 | 0.40 | 0.17 | 0.14 | 0.94 | 0.71 | 0.66 | 0.85 | 0.0101 | 0.0327 | 0.2281 | 0.2482 | 7.5798 | 0.0246 | 3.6188 | 0.0190 |
| N47 | 0.74 | 0.57 | 0.26 | 0.29 | 0.20 | 0.29 | 0.22 | 0.92 | 0.24 | 0.37 | 0.39 | 0.16 | 0.22 | 1.04 | 0.72 | 0.65 | 0.86 | 0.0090 | 0.0374 | 0.1870 | 0.2373 | 8.4565 | 0.0246 | 3.7802 | 0.0203 |
| Label | HPLC-UV | | | | | | | | | | | | | | HPLC-ELSD | | | q^1^HNMR | | | | | | | |
|  | F1 | F2 | F3 | F4 | F5 | F6 | F7 | F8 | F9 | F10 | F11 | F12 | F13 | F14 | F15 | F16 | F17 | F18 | F19 | F20 | F21 | F22 | F23 | F24 | F25 |
| N48 | 0.78 | 0.59 | 0.29 | 0.32 | 0.21 | 0.25 | 0.24 | 0.92 | 0.34 | 0.40 | 0.45 | 0.18 | 0.23 | 1.08 | 0.73 | 0.65 | 0.86 | 0.0091 | 0.0406 | 0.1897 | 0.2110 | 7.7686 | 0.0225 | 3.7803 | 0.0167 |
| N49 | 0.74 | 0.57 | 0.27 | 0.31 | 0.20 | 0.27 | 0.23 | 0.88 | 0.27 | 0.39 | 0.43 | 0.17 | 0.21 | 1.02 | 0.72 | 0.65 | 0.85 | 0.0088 | 0.0434 | 0.2063 | 0.2259 | 7.9848 | 0.0204 | 3.7304 | 0.0200 |
| N50 | 0.82 | 0.65 | 0.31 | 0.36 | 0.23 | 0.33 | 0.26 | 1.02 | 0.30 | 0.42 | 0.42 | 0.19 | 0.22 | 1.16 | 0.73 | 0.66 | 0.86 | 0.0095 | 0.0406 | 0.2194 | 0.2513 | 8.5424 | 0.0226 | 3.8192 | 0.0269 |
| N51 | 0.87 | 0.64 | 0.31 | 0.27 | 0.20 | 0.30 | 0.23 | 0.85 | 0.27 | 0.40 | 0.42 | 0.17 | 0.21 | 1.01 | 0.73 | 0.65 | 0.88 | 0.0135 | 0.0587 | 0.2085 | 0.2228 | 7.7622 | 0.0249 | 3.6305 | 0.0214 |
| N52 | 1.06 | 0.76 | 0.35 | 0.32 | 0.24 | 0.33 | 0.27 | 1.03 | 0.35 | 0.47 | 0.28 | 0.19 | 0.23 | 1.21 | 0.75 | 0.68 | 0.90 | 0.0108 | 0.0341 | 0.1969 | 0.2110 | 7.8747 | 0.0240 | 3.9242 | 0.0185 |
| A1 | 0.49 | 0.70 | 0.33 | 0.33 | 0.21 | 0.31 | 0.25 | 0.91 | 0.25 | 0.44 | 0.44 | 0.19 | 0.25 | 1.04 | 0.64 | 0.61 | 0.82 | 0.0096 | 0.0330 | 0.2887 | 0.3568 | 8.0725 | 0.0331 | 4.6301 | 0.0171 |
| A2 | 0.47 | 0.59 | 0.29 | 0.28 | 0.19 | 0.31 | 0.23 | 0.76 | 0.28 | 0.39 | 0.40 | 0.18 | 0.22 | 0.88 | 0.64 | 0.61 | 0.81 | 0.0089 | 0.0712 | 0.3054 | 0.3275 | 7.6865 | 0.0370 | 4.6563 | 0.0167 |
| A3 | 0.56 | 0.60 | 0.30 | 0.27 | 0.19 | 0.28 | 0.23 | 0.85 | 0.37 | 0.42 | 0.44 | 0.17 | 0.14 | 0.96 | 0.64 | 0.60 | 0.82 | 0.0053 | 0.0744 | 0.3051 | 0.3486 | 7.0587 | 0.0362 | 4.6072 | 0.0148 |
| A4 | 0.57 | 0.63 | 0.32 | 0.28 | 0.19 | 0.28 | 0.29 | 0.85 | 0.26 | 0.45 | 0.45 | 0.18 | 0.14 | 0.99 | 0.64 | 0.61 | 0.81 | 0.0067 | 0.0892 | 0.2927 | 0.3237 | 7.0066 | 0.0337 | 4.5060 | 0.0139 |
| A5 | 0.52 | 0.59 | 0.30 | 0.26 | 0.18 | 0.27 | 0.22 | 0.79 | 0.25 | 0.42 | 0.38 | 0.16 | 0.21 | 0.91 | 0.64 | 0.61 | 0.81 | 0.0111 | 0.0787 | 0.2824 | 0.3161 | 7.0553 | 0.0324 | 4.6125 | 0.0158 |
| A6 | 0.55 | 0.61 | 0.32 | 0.28 | 0.18 | 0.26 | 0.28 | 0.80 | 0.27 | 0.44 | 0.44 | 0.17 | 0.14 | 0.94 | 0.64 | 0.61 | 0.81 | 0.0087 | 0.0739 | 0.3016 | 0.3457 | 7.0599 | 0.0367 | 4.8568 | 0.0157 |
| A7 | 0.40 | 0.65 | 0.33 | 0.30 | 0.20 | 0.28 | 0.29 | 0.86 | 0.28 | 0.45 | 0.47 | 0.18 | 0.23 | 1.03 | 0.65 | 0.61 | 0.82 | 0.0067 | 0.0767 | 0.2634 | 0.2850 | 8.1269 | 0.0312 | 3.8310 | 0.0137 |
| A8 | 0.55 | 0.63 | 0.30 | 0.33 | 0.21 | 0.25 | 0.26 | 0.82 | 0.44 | 0.45 | 0.46 | 0.19 | 0.21 | 0.99 | 0.62 | 0.62 | 0.81 | 0.0062 | 0.0670 | 0.2626 | 0.2656 | 7.7183 | 0.0324 | 5.0860 | 0.0144 |
| A9 | 0.58 | 0.65 | 0.31 | 0.34 | 0.22 | 0.26 | 0.26 | 0.86 | 0.44 | 0.46 | 0.48 | 0.19 | 0.21 | 1.04 | 0.63 | 0.61 | 0.81 | 0.0083 | 0.0702 | 0.2814 | 0.2983 | 6.9757 | 0.0327 | 4.5997 | 0.0150 |
| A10 | 0.58 | 0.69 | 0.32 | 0.35 | 0.24 | 0.27 | 0.27 | 0.90 | 0.36 | 0.48 | 0.50 | 0.19 | 0.22 | 1.09 | 0.61 | 0.62 | 0.77 | 0.0079 | 0.0759 | 0.2470 | 0.2888 | 6.9843 | 0.0325 | 4.3608 | 0.0167 |
| A11 | 0.58 | 0.67 | 0.33 | 0.39 | 0.22 | 0.26 | 0.27 | 0.87 | 0.45 | 0.48 | 0.49 | 0.20 | 0.16 | 1.05 | 0.67 | 0.63 | 0.82 | 0.0055 | 0.0587 | 0.2726 | 0.3085 | 6.6642 | 0.0337 | 4.5515 | 0.0175 |
| A12 | 0.55 | 0.64 | 0.31 | 0.34 | 0.21 | 0.25 | 0.26 | 0.85 | 0.40 | 0.44 | 0.46 | 0.18 | 0.15 | 1.00 | 0.67 | 0.63 | 0.82 | 0.0035 | 0.0572 | 0.2855 | 0.3356 | 7.5934 | 0.0354 | 5.1787 | 0.0161 |
| A13 | 0.50 | 0.65 | 0.30 | 0.33 | 0.22 | 0.24 | 0.26 | 0.85 | 0.29 | 0.43 | 0.45 | 0.18 | 0.24 | 1.00 | 0.67 | 0.64 | 0.83 | 0.0093 | 0.1029 | 0.3227 | 0.4047 | 6.7750 | 0.0355 | 4.6838 | 0.0126 |
| A14 | 0.46 | 0.55 | 0.28 | 0.30 | 0.19 | 0.23 | 0.23 | 0.70 | 0.38 | 0.41 | 0.41 | 0.17 | 0.14 | 0.85 | 0.66 | 0.64 | 0.81 | 0.0097 | 0.0809 | 0.2651 | 0.2989 | 7.3538 | 0.0340 | 4.4614 | 0.0171 |
| A15 | 0.34 | 0.48 | 0.19 | 0.27 | 0.14 | 0.29 | 0.22 | 0.68 | 0.39 | 0.35 | 0.35 | 0.16 | 0.14 | 0.76 | 0.68 | 0.65 | 0.83 | 0.0067 | 0.0778 | 0.3199 | 0.4053 | 7.3858 | 0.0365 | 4.7037 | 0.0126 |
| A16 | 0.39 | 0.53 | 0.20 | 0.28 | 0.15 | 0.22 | 0.22 | 0.79 | 0.26 | 0.35 | 0.38 | 0.16 | 0.14 | 0.87 | 0.68 | 0.64 | 0.82 | 0.0087 | 0.1043 | 0.3248 | 0.3418 | 8.1874 | 0.0347 | 4.9734 | 0.0127 |
| A17 | 0.38 | 0.54 | 0.21 | 0.27 | 0.15 | 0.28 | 0.26 | 0.81 | 0.27 | 0.34 | 0.38 | 0.16 | 0.14 | 0.88 | 0.67 | 0.65 | 0.82 | 0.0055 | 0.0679 | 0.2789 | 0.3476 | 8.0936 | 0.0318 | 4.8041 | 0.0144 |
| A18 | 0.44 | 0.65 | 0.26 | 0.38 | 0.21 | 0.27 | 0.28 | 0.92 | 0.40 | 0.44 | 0.47 | 0.21 | 0.18 | 1.01 | 0.66 | 0.64 | 0.82 | 0.0084 | 0.0450 | 0.3305 | 0.3692 | 7.3997 | 0.0341 | 4.8905 | 0.0171 |
| A19 | 0.38 | 0.56 | 0.23 | 0.33 | 0.16 | 0.27 | 0.24 | 1.00 | 0.24 | 0.36 | 0.42 | 0.18 | 0.19 | 0.90 | 0.68 | 0.65 | 0.82 | 0.0089 | 0.0648 | 0.3683 | 0.3966 | 8.0740 | 0.0290 | 4.9505 | 0.0150 |
| Label | HPLC-UV | | | | | | | | | | | | | | HPLC-ELSD | | | q^1^HNMR | | | | | | | |
|  | F1 | F2 | F3 | F4 | F5 | F6 | F7 | F8 | F9 | F10 | F11 | F12 | F13 | F14 | F15 | F16 | F17 | F18 | F19 | F20 | F21 | F22 | F23 | F24 | F25 |
| A20 | 0.34 | 0.67 | 0.29 | 0.46 | 0.22 | 0.26 | 0.29 | 0.88 | 0.47 | 0.44 | 0.45 | 0.21 | 0.17 | 0.98 | 0.67 | 0.63 | 0.81 | 0.0105 | 0.0873 | 0.2685 | 0.3171 | 8.5554 | 0.0350 | 4.6376 | 0.0132 |
| A21 | 0.48 | 0.64 | 0.28 | 0.44 | 0.22 | 0.25 | 0.27 | 0.85 | 0.44 | 0.44 | 0.44 | 0.20 | 0.17 | 0.95 | 0.66 | 0.64 | 0.81 | 0.0055 | 0.0648 | 0.2584 | 0.3022 | 7.4950 | 0.0305 | 4.3147 | 0.0173 |
| A22 | 0.54 | 0.61 | 0.30 | 0.37 | 0.21 | 0.32 | 0.25 | 0.96 | 0.33 | 0.43 | 0.47 | 0.18 | 0.17 | 1.10 | 0.71 | 0.68 | 0.84 | 0.0074 | 0.0772 | 0.3229 | 0.3578 | 7.5941 | 0.0311 | 4.8078 | 0.0149 |
| A23 | 0.54 | 0.61 | 0.30 | 0.37 | 0.21 | 0.32 | 0.24 | 0.96 | 0.34 | 0.47 | 0.47 | 0.18 | 0.17 | 1.11 | 0.68 | 0.63 | 0.82 | 0.0079 | 0.0774 | 0.2997 | 0.3254 | 7.6725 | 0.0297 | 4.5900 | 0.0140 |
| A24 | 0.53 | 0.67 | 0.33 | 0.42 | 0.26 | 0.30 | 0.28 | 1.03 | 0.49 | 0.51 | 0.53 | 0.22 | 0.14 | 1.23 | 0.68 | 0.64 | 0.83 | 0.0065 | 0.0606 | 0.3073 | 0.3320 | 7.8777 | 0.0318 | 4.4689 | 0.0116 |
| A25 | 0.65 | 0.69 | 0.34 | 0.32 | 0.22 | 0.36 | 0.36 | 0.91 | 0.36 | 0.49 | 0.50 | 0.17 | 0.16 | 1.10 | 0.68 | 0.63 | 0.84 | 0.0098 | 0.0821 | 0.2564 | 0.3320 | 7.5026 | 0.0326 | 4.9512 | 0.0172 |
| A26 | 0.77 | 0.83 | 0.39 | 0.37 | 0.26 | 0.38 | 0.29 | 1.10 | 0.37 | 0.57 | 0.59 | 0.22 | 0.18 | 1.32 | 0.68 | 0.62 | 0.83 | 0.0069 | 0.0813 | 0.3179 | 0.3614 | 7.2860 | 0.0364 | 4.4579 | 0.0173 |

**Table S10.** Trainset based on fused feature table

| Label | F1 | F2 | F3 | F4 | F5 | F6 | F7 | F8 | F9 | F10 | F11 | F12 | F13 | F14 | F15 | F16 | F17 | F18 | F19 | F20 | F21 | F22 | F23 | F24 | F25 |
| --- | --- | --- | --- | --- | --- | --- | --- | --- | --- | --- | --- | --- | --- | --- | --- | --- | --- | --- | --- | --- | --- | --- | --- | --- | --- |
| N10 | 0.71 | 0.66 | 0.29 | 0.42 | 0.20 | 0.26 | 0.28 | 1.10 | 0.36 | 0.44 | 0.30 | 0.18 | 0.34 | 1.03 | 0.70 | 0.71 | 0.88 | 0.0108 | 0.0588 | 0.2373 | 0.2619 | 9.5460 | 0.0272 | 4.8093 | 0.0214 |
| N47 | 0.74 | 0.57 | 0.26 | 0.29 | 0.20 | 0.29 | 0.22 | 0.92 | 0.24 | 0.37 | 0.39 | 0.16 | 0.22 | 1.04 | 0.72 | 0.65 | 0.86 | 0.0090 | 0.0374 | 0.1870 | 0.2373 | 8.4565 | 0.0246 | 3.7802 | 0.0203 |
| N5 | 0.53 | 0.54 | 0.24 | 0.33 | 0.17 | 0.21 | 0.26 | 0.89 | 0.31 | 0.31 | 0.39 | 0.24 | 0.29 | 0.84 | 0.70 | 0.72 | 0.86 | 0.0105 | 0.0748 | 0.2164 | 0.2804 | 9.3257 | 0.0286 | 4.8053 | 0.0194 |
| N9 | 0.68 | 0.64 | 0.29 | 0.42 | 0.21 | 0.23 | 0.27 | 0.91 | 0.38 | 0.39 | 0.50 | 0.17 | 0.34 | 1.01 | 0.70 | 0.72 | 0.87 | 0.0087 | 0.0481 | 0.2068 | 0.2392 | 8.9532 | 0.0251 | 4.2630 | 0.0204 |
| N18 | 0.75 | 0.59 | 0.30 | 0.32 | 0.22 | 0.30 | 0.36 | 1.06 | 0.34 | 0.46 | 0.58 | 0.16 | 0.31 | 1.01 | 0.71 | 0.71 | 0.88 | 0.0108 | 0.0676 | 0.2388 | 0.2832 | 10.1444 | 0.0262 | 4.4942 | 0.0312 |
| N8 | 0.72 | 0.72 | 0.29 | 0.44 | 0.22 | 0.24 | 0.39 | 1.00 | 0.35 | 0.46 | 0.33 | 0.18 | 0.36 | 1.09 | 0.70 | 0.71 | 0.88 | 0.0103 | 0.0538 | 0.2232 | 0.2520 | 9.2603 | 0.0270 | 4.3911 | 0.0235 |
| N32 | 0.91 | 0.66 | 0.31 | 0.33 | 0.22 | 0.28 | 0.26 | 0.86 | 0.34 | 0.42 | 0.43 | 0.17 | 0.14 | 1.04 | 0.71 | 0.70 | 0.90 | 0.0139 | 0.0517 | 0.2465 | 0.2559 | 9.4124 | 0.0285 | 4.3085 | 0.0248 |
| N6 | 0.76 | 0.70 | 0.30 | 0.48 | 0.23 | 0.34 | 0.31 | 1.14 | 0.38 | 0.45 | 0.47 | 0.28 | 0.40 | 1.07 | 0.70 | 0.71 | 0.87 | 0.0118 | 0.0507 | 0.2021 | 0.2497 | 9.2418 | 0.0264 | 4.4207 | 0.0280 |
| N12 | 0.70 | 0.60 | 0.29 | 0.39 | 0.22 | 0.31 | 0.28 | 1.06 | 0.39 | 0.21 | 0.30 | 0.17 | 0.32 | 0.94 | 0.69 | 0.71 | 0.87 | 0.0106 | 0.0548 | 0.2154 | 0.2442 | 9.2853 | 0.0259 | 4.4426 | 0.0202 |
| N27 | 1.08 | 0.61 | 0.30 | 0.26 | 0.18 | 0.28 | 0.22 | 0.96 | 0.30 | 0.41 | 0.44 | 0.26 | 0.30 | 0.96 | 0.72 | 0.70 | 0.89 | 0.0147 | 0.0553 | 0.2524 | 0.2578 | 9.7716 | 0.0289 | 4.6202 | 0.0256 |
| N25 | 1.03 | 0.64 | 0.31 | 0.28 | 0.19 | 0.29 | 0.23 | 0.93 | 0.29 | 0.40 | 0.49 | 0.26 | 0.31 | 0.93 | 0.71 | 0.71 | 0.89 | 0.0123 | 0.0587 | 0.2327 | 0.2591 | 9.0637 | 0.0299 | 4.3122 | 0.0297 |
| N28 | 1.01 | 0.57 | 0.29 | 0.24 | 0.17 | 0.28 | 0.21 | 0.88 | 0.29 | 0.35 | 0.37 | 0.22 | 0.16 | 0.84 | 0.72 | 0.71 | 0.89 | 0.0140 | 0.0689 | 0.2492 | 0.2572 | 9.5167 | 0.0290 | 4.7368 | 0.0251 |
| N7 | 0.77 | 0.72 | 0.30 | 0.45 | 0.23 | 0.25 | 0.32 | 1.14 | 0.34 | 0.28 | 0.44 | 0.21 | 0.30 | 1.04 | 0.70 | 0.71 | 0.88 | 0.0130 | 0.0512 | 0.2146 | 0.2572 | 9.1037 | 0.0271 | 4.4349 | 0.0235 |
| N21 | 1.08 | 0.63 | 0.31 | 0.28 | 0.18 | 0.31 | 0.23 | 0.92 | 0.33 | 0.45 | 0.41 | 0.21 | 0.31 | 0.91 | 0.71 | 0.70 | 0.89 | 0.0130 | 0.0676 | 0.2289 | 0.2561 | 9.9296 | 0.0321 | 4.4221 | 0.0213 |
| N44 | 0.55 | 0.52 | 0.21 | 0.28 | 0.15 | 0.25 | 0.23 | 0.92 | 0.28 | 0.32 | 0.38 | 0.15 | 0.21 | 0.84 | 0.72 | 0.71 | 0.88 | 0.0103 | 0.0546 | 0.2104 | 0.2246 | 7.7393 | 0.0233 | 3.8164 | 0.0234 |
| N48 | 0.78 | 0.59 | 0.29 | 0.32 | 0.21 | 0.25 | 0.24 | 0.92 | 0.34 | 0.40 | 0.45 | 0.18 | 0.23 | 1.08 | 0.73 | 0.65 | 0.86 | 0.0091 | 0.0406 | 0.1897 | 0.2110 | 7.7686 | 0.0225 | 3.7803 | 0.0167 |
| N39 | 0.75 | 0.56 | 0.28 | 0.29 | 0.19 | 0.22 | 0.23 | 0.71 | 0.26 | 0.38 | 0.37 | 0.15 | 0.20 | 0.85 | 0.70 | 0.71 | 0.90 | 0.0112 | 0.0439 | 0.2021 | 0.2222 | 8.1949 | 0.0246 | 3.4942 | 0.0232 |
| N17 | 0.67 | 0.57 | 0.26 | 0.29 | 0.19 | 0.25 | 0.24 | 0.96 | 0.24 | 0.31 | 0.35 | 0.18 | 0.22 | 0.89 | 0.71 | 0.71 | 0.88 | 0.0111 | 0.0574 | 0.2574 | 0.2829 | 8.9869 | 0.0272 | 4.3625 | 0.0240 |
| N22 | 0.99 | 0.59 | 0.30 | 0.26 | 0.17 | 0.27 | 0.22 | 0.86 | 0.31 | 0.43 | 0.39 | 0.25 | 0.30 | 0.85 | 0.71 | 0.71 | 0.89 | 0.0105 | 0.0673 | 0.2178 | 0.2376 | 9.8017 | 0.0299 | 4.2847 | 0.3871 |
| N52 | 1.06 | 0.76 | 0.35 | 0.32 | 0.24 | 0.33 | 0.27 | 1.03 | 0.35 | 0.47 | 0.28 | 0.19 | 0.23 | 1.21 | 0.75 | 0.68 | 0.90 | 0.0108 | 0.0341 | 0.1969 | 0.2110 | 7.8747 | 0.0240 | 3.9242 | 0.0185 |
| N30 | 1.14 | 0.64 | 0.32 | 0.27 | 0.19 | 0.29 | 0.23 | 1.00 | 0.33 | 0.47 | 0.54 | 0.27 | 0.32 | 1.01 | 0.72 | 0.71 | 0.89 | 0.0136 | 0.0626 | 0.2500 | 0.2534 | 9.6715 | 0.0294 | 4.4907 | 0.0243 |
| N23 | 1.10 | 0.62 | 0.31 | 0.27 | 0.19 | 0.29 | 0.23 | 0.99 | 0.34 | 0.45 | 0.52 | 0.26 | 0.28 | 0.98 | 0.70 | 0.72 | 0.89 | 0.0121 | 0.0525 | 0.2270 | 0.2497 | 9.0301 | 0.0322 | 4.6807 | 0.0247 |
| N36 | 0.88 | 0.70 | 0.32 | 0.34 | 0.22 | 0.33 | 0.26 | 0.89 | 0.27 | 0.43 | 0.43 | 0.18 | 0.23 | 1.06 | 0.70 | 0.69 | 0.90 | 0.0110 | 0.0621 | 0.2152 | 0.2498 | 10.0181 | 0.0290 | 4.5052 | 0.0371 |
| N24 | 1.03 | 0.66 | 0.30 | 0.28 | 0.20 | 0.28 | 0.24 | 0.97 | 0.28 | 0.39 | 0.42 | 0.26 | 0.31 | 0.97 | 0.72 | 0.71 | 0.90 | 0.0121 | 0.0504 | 0.2169 | 0.2247 | 9.7187 | 0.0235 | 4.5214 | 0.0217 |
| Label | F1 | F2 | F3 | F4 | F5 | F6 | F7 | F8 | F9 | F10 | F11 | F12 | F13 | F14 | F15 | F16 | F17 | F18 | F19 | F20 | F21 | F22 | F23 | F24 | F25 |
| N4 | 0.44 | 0.54 | 0.22 | 0.26 | 0.15 | 0.28 | 0.26 | 1.11 | 0.22 | 0.37 | 0.45 | 0.23 | 0.30 | 0.95 | 0.72 | 0.70 | 0.88 | 0.0102 | 0.0569 | 0.2397 | 0.3036 | 10.0874 | 0.0224 | 4.3627 | 0.0225 |
| N34 | 0.97 | 0.72 | 0.33 | 0.35 | 0.23 | 0.27 | 0.27 | 0.93 | 0.31 | 0.45 | 0.46 | 0.18 | 0.24 | 1.13 | 0.70 | 0.69 | 0.91 | 0.0124 | 0.0543 | 0.2178 | 0.2330 | 9.8112 | 0.0280 | 4.3600 | 0.0239 |
| N19 | 0.79 | 0.61 | 0.30 | 0.27 | 0.19 | 0.31 | 0.24 | 0.93 | 0.28 | 0.41 | 0.46 | 0.21 | 0.29 | 0.90 | 0.72 | 0.71 | 0.88 | 0.0118 | 0.0577 | 0.2473 | 0.2965 | 9.5327 | 0.0274 | 4.3083 | 0.0240 |
| N14 | 0.76 | 0.62 | 0.31 | 0.29 | 0.24 | 0.38 | 0.29 | 1.14 | 0.38 | 0.42 | 0.47 | 0.16 | 0.34 | 1.08 | 0.72 | 0.70 | 0.89 | 0.0119 | 0.0548 | 0.2545 | 0.2869 | 9.3135 | 0.0275 | 4.4723 | 0.0237 |
| N45 | 0.76 | 0.67 | 0.28 | 0.41 | 0.21 | 0.28 | 0.27 | 0.86 | 0.34 | 0.38 | 0.38 | 0.16 | 0.24 | 0.97 | 0.70 | 0.66 | 0.85 | 0.0096 | 0.0427 | 0.2468 | 0.2902 | 7.9533 | 0.0208 | 3.9882 | 0.0220 |
| N37 | 0.80 | 0.69 | 0.31 | 0.32 | 0.22 | 0.32 | 0.26 | 0.88 | 0.25 | 0.40 | 0.41 | 0.17 | 0.22 | 1.03 | 0.70 | 0.69 | 0.91 | 0.0132 | 0.0481 | 0.1951 | 0.2147 | 7.7004 | 0.0251 | 3.8655 | 0.0248 |
| N13 | 0.82 | 0.65 | 0.29 | 0.38 | 0.21 | 0.28 | 0.27 | 1.08 | 0.33 | 0.36 | 0.42 | 0.19 | 0.25 | 0.97 | 0.70 | 0.71 | 0.88 | 0.0125 | 0.0585 | 0.2280 | 0.2682 | 10.3996 | 0.0274 | 4.4420 | 0.0204 |
| N26 | 1.14 | 0.65 | 0.31 | 0.27 | 0.19 | 0.30 | 0.24 | 1.02 | 0.33 | 0.47 | 0.54 | 0.23 | 0.31 | 1.02 | 0.72 | 0.71 | 0.89 | 0.0106 | 0.0629 | 0.2378 | 0.2675 | 9.9687 | 0.0306 | 4.6799 | 0.0206 |
| N42 | 0.52 | 0.51 | 0.20 | 0.20 | 0.14 | 0.23 | 0.19 | 0.75 | 0.26 | 0.31 | 0.32 | 0.14 | 0.21 | 0.84 | 0.71 | 0.72 | 0.90 | 0.0087 | 0.0434 | 0.2255 | 0.2566 | 7.9119 | 0.0250 | 3.6067 | 0.0155 |
| N33 | 0.91 | 0.69 | 0.32 | 0.35 | 0.23 | 0.30 | 0.27 | 0.90 | 0.32 | 0.44 | 0.44 | 0.18 | 0.23 | 1.09 | 0.70 | 0.70 | 0.90 | 0.0133 | 0.0639 | 0.2310 | 0.2395 | 9.3757 | 0.0295 | 4.6108 | 0.0234 |
| N41 | 0.57 | 0.50 | 0.19 | 0.22 | 0.14 | 0.21 | 0.21 | 0.74 | 0.26 | 0.32 | 0.35 | 0.15 | 0.21 | 0.83 | 0.74 | 0.70 | 0.88 | 0.0092 | 0.0414 | 0.1782 | 0.1824 | 7.8149 | 0.0241 | 3.7042 | 0.0196 |
| N31 | 1.21 | 0.67 | 0.33 | 0.27 | 0.20 | 0.31 | 0.31 | 1.05 | 0.34 | 0.46 | 0.57 | 0.33 | 0.33 | 1.06 | 0.72 | 0.70 | 0.90 | 0.0139 | 0.0791 | 0.2775 | 0.2524 | 9.7310 | 0.0297 | 4.5463 | 0.0294 |
| A5 | 0.52 | 0.59 | 0.30 | 0.26 | 0.18 | 0.27 | 0.22 | 0.79 | 0.25 | 0.42 | 0.38 | 0.16 | 0.21 | 0.91 | 0.64 | 0.61 | 0.81 | 0.0111 | 0.0787 | 0.2824 | 0.3161 | 7.0553 | 0.0324 | 4.6125 | 0.0158 |
| A7 | 0.40 | 0.65 | 0.33 | 0.30 | 0.20 | 0.28 | 0.29 | 0.86 | 0.28 | 0.45 | 0.47 | 0.18 | 0.23 | 1.03 | 0.65 | 0.61 | 0.82 | 0.0067 | 0.0767 | 0.2634 | 0.2850 | 8.1269 | 0.0312 | 3.8310 | 0.0137 |
| A17 | 0.38 | 0.54 | 0.21 | 0.27 | 0.15 | 0.28 | 0.26 | 0.81 | 0.27 | 0.34 | 0.38 | 0.16 | 0.14 | 0.88 | 0.67 | 0.65 | 0.82 | 0.0055 | 0.0679 | 0.2789 | 0.3476 | 8.0936 | 0.0318 | 4.8041 | 0.0144 |
| A4 | 0.57 | 0.63 | 0.32 | 0.28 | 0.19 | 0.28 | 0.29 | 0.85 | 0.26 | 0.45 | 0.45 | 0.18 | 0.14 | 0.99 | 0.64 | 0.61 | 0.81 | 0.0067 | 0.0892 | 0.2927 | 0.3237 | 7.0066 | 0.0337 | 4.5060 | 0.0139 |
| A1 | 0.49 | 0.70 | 0.33 | 0.33 | 0.21 | 0.31 | 0.25 | 0.91 | 0.25 | 0.44 | 0.44 | 0.19 | 0.25 | 1.04 | 0.64 | 0.61 | 0.82 | 0.0096 | 0.0330 | 0.2887 | 0.3568 | 8.0725 | 0.0331 | 4.6301 | 0.0171 |
| A2 | 0.47 | 0.59 | 0.29 | 0.28 | 0.19 | 0.31 | 0.23 | 0.76 | 0.28 | 0.39 | 0.40 | 0.18 | 0.22 | 0.88 | 0.64 | 0.61 | 0.81 | 0.0089 | 0.0712 | 0.3054 | 0.3275 | 7.6865 | 0.0370 | 4.6563 | 0.0167 |
| A6 | 0.55 | 0.61 | 0.32 | 0.28 | 0.18 | 0.26 | 0.28 | 0.80 | 0.27 | 0.44 | 0.44 | 0.17 | 0.14 | 0.94 | 0.64 | 0.61 | 0.81 | 0.0087 | 0.0739 | 0.3016 | 0.3457 | 7.0599 | 0.0367 | 4.8568 | 0.0157 |
| A22 | 0.54 | 0.61 | 0.30 | 0.37 | 0.21 | 0.32 | 0.25 | 0.96 | 0.33 | 0.43 | 0.47 | 0.18 | 0.17 | 1.10 | 0.71 | 0.68 | 0.84 | 0.0074 | 0.0772 | 0.3229 | 0.3578 | 7.5941 | 0.0311 | 4.8078 | 0.0149 |
| A14 | 0.46 | 0.55 | 0.28 | 0.30 | 0.19 | 0.23 | 0.23 | 0.70 | 0.38 | 0.41 | 0.41 | 0.17 | 0.14 | 0.85 | 0.66 | 0.64 | 0.81 | 0.0097 | 0.0809 | 0.2651 | 0.2989 | 7.3538 | 0.0340 | 4.4614 | 0.0171 |
| A20 | 0.34 | 0.67 | 0.29 | 0.46 | 0.22 | 0.26 | 0.29 | 0.88 | 0.47 | 0.44 | 0.45 | 0.21 | 0.17 | 0.98 | 0.67 | 0.63 | 0.81 | 0.0105 | 0.0873 | 0.2685 | 0.3171 | 8.5554 | 0.0350 | 4.6376 | 0.0132 |
| A19 | 0.38 | 0.56 | 0.23 | 0.33 | 0.16 | 0.27 | 0.24 | 1.00 | 0.24 | 0.36 | 0.42 | 0.18 | 0.19 | 0.90 | 0.68 | 0.65 | 0.82 | 0.0089 | 0.0648 | 0.3683 | 0.3966 | 8.0740 | 0.0290 | 4.9505 | 0.0150 |
| A9 | 0.58 | 0.65 | 0.31 | 0.34 | 0.22 | 0.26 | 0.26 | 0.86 | 0.44 | 0.46 | 0.48 | 0.19 | 0.21 | 1.04 | 0.63 | 0.61 | 0.81 | 0.0083 | 0.0702 | 0.2814 | 0.2983 | 6.9757 | 0.0327 | 4.5997 | 0.0150 |
| A13 | 0.50 | 0.65 | 0.30 | 0.33 | 0.22 | 0.24 | 0.26 | 0.85 | 0.29 | 0.43 | 0.45 | 0.18 | 0.24 | 1.00 | 0.67 | 0.64 | 0.83 | 0.0093 | 0.1029 | 0.3227 | 0.4047 | 6.7750 | 0.0355 | 4.6838 | 0.0126 |
| Label | F1 | F2 | F3 | F4 | F5 | F6 | F7 | F8 | F9 | F10 | F11 | F12 | F13 | F14 | F15 | F16 | F17 | F18 | F19 | F20 | F21 | F22 | F23 | F24 | F25 |
| A11 | 0.58 | 0.67 | 0.33 | 0.39 | 0.22 | 0.26 | 0.27 | 0.87 | 0.45 | 0.48 | 0.49 | 0.20 | 0.16 | 1.05 | 0.67 | 0.63 | 0.82 | 0.0055 | 0.0587 | 0.2726 | 0.3085 | 6.6642 | 0.0337 | 4.5515 | 0.0175 |
| A21 | 0.48 | 0.64 | 0.28 | 0.44 | 0.22 | 0.25 | 0.27 | 0.85 | 0.44 | 0.44 | 0.44 | 0.20 | 0.17 | 0.95 | 0.66 | 0.64 | 0.81 | 0.0055 | 0.0648 | 0.2584 | 0.3022 | 7.4950 | 0.0305 | 4.3147 | 0.0173 |
| A12 | 0.55 | 0.64 | 0.31 | 0.34 | 0.21 | 0.25 | 0.26 | 0.85 | 0.40 | 0.44 | 0.46 | 0.18 | 0.15 | 1.00 | 0.67 | 0.63 | 0.82 | 0.0035 | 0.0572 | 0.2855 | 0.3356 | 7.5934 | 0.0354 | 5.1787 | 0.0161 |
| A16 | 0.39 | 0.53 | 0.20 | 0.28 | 0.15 | 0.22 | 0.22 | 0.79 | 0.26 | 0.35 | 0.38 | 0.16 | 0.14 | 0.87 | 0.68 | 0.64 | 0.82 | 0.0087 | 0.1043 | 0.3248 | 0.3418 | 8.1874 | 0.0347 | 4.9734 | 0.0127 |
| A15 | 0.34 | 0.48 | 0.19 | 0.27 | 0.14 | 0.29 | 0.22 | 0.68 | 0.39 | 0.35 | 0.35 | 0.16 | 0.14 | 0.76 | 0.68 | 0.65 | 0.83 | 0.0067 | 0.0778 | 0.3199 | 0.4053 | 7.3858 | 0.0365 | 4.7037 | 0.0126 |

**Table S11.** Testset based on fused feature table

| Label | F1 | F2 | F3 | F4 | F5 | F6 | F7 | F8 | F9 | F10 | F11 | F12 | F13 | F14 | F15 | F16 | F17 | F18 | F19 | F20 | F21 | F22 | F23 | F24 | F25 |
| --- | --- | --- | --- | --- | --- | --- | --- | --- | --- | --- | --- | --- | --- | --- | --- | --- | --- | --- | --- | --- | --- | --- | --- | --- | --- |
| N16 | 0.56 | 0.47 | 0.25 | 0.22 | 0.19 | 0.26 | 0.23 | 0.85 | 0.25 | 0.38 | 0.46 | 0.13 | 0.31 | 0.79 | 0.71 | 0.72 | 0.88 | 0.0094 | 0.0600 | 0.2296 | 0.3076 | 9.1029 | 0.0263 | 4.6707 | 0.0279 |
| N40 | 0.55 | 0.53 | 0.20 | 0.26 | 0.13 | 0.24 | 0.23 | 0.72 | 0.27 | 0.34 | 0.32 | 0.15 | 0.21 | 0.81 | 0.71 | 0.72 | 0.90 | 0.0090 | 0.0468 | 0.1756 | 0.1891 | 7.6511 | 0.0230 | 3.5740 | 0.0247 |
| N46 | 0.76 | 0.64 | 0.27 | 0.40 | 0.20 | 0.27 | 0.26 | 0.84 | 0.27 | 0.38 | 0.40 | 0.17 | 0.14 | 0.94 | 0.71 | 0.66 | 0.85 | 0.0101 | 0.0327 | 0.2281 | 0.2482 | 7.5798 | 0.0246 | 3.6188 | 0.0190 |
| N49 | 0.74 | 0.57 | 0.27 | 0.31 | 0.20 | 0.27 | 0.23 | 0.88 | 0.27 | 0.39 | 0.43 | 0.17 | 0.21 | 1.02 | 0.72 | 0.65 | 0.85 | 0.0088 | 0.0434 | 0.2063 | 0.2259 | 7.9848 | 0.0204 | 3.7304 | 0.0200 |
| N38 | 0.81 | 0.72 | 0.32 | 0.34 | 0.23 | 0.30 | 0.27 | 0.93 | 0.27 | 0.43 | 0.44 | 0.18 | 0.23 | 1.09 | 0.70 | 0.68 | 0.90 | 0.0096 | 0.0421 | 0.1999 | 0.2128 | 8.0776 | 0.0245 | 3.6674 | 0.0263 |
| N2 | 0.58 | 0.63 | 0.24 | 0.29 | 0.18 | 0.33 | 0.28 | 1.07 | 0.32 | 0.27 | 0.40 | 0.20 | 0.27 | 0.96 | 0.72 | 0.69 | 0.87 | 0.0140 | 0.0561 | 0.2829 | 0.3149 | 9.9845 | 0.0285 | 4.6020 | 0.0312 |
| N35 | 0.93 | 0.71 | 0.33 | 0.36 | 0.23 | 0.30 | 0.27 | 0.91 | 0.33 | 0.45 | 0.45 | 0.18 | 0.24 | 1.09 | 0.69 | 0.69 | 0.90 | 0.0132 | 0.0788 | 0.2018 | 0.2371 | 8.7042 | 0.0282 | 4.1748 | 0.0179 |
| N20 | 1.00 | 0.68 | 0.33 | 0.29 | 0.19 | 0.30 | 0.24 | 1.00 | 0.34 | 0.36 | 0.39 | 0.24 | 0.27 | 0.95 | 0.72 | 0.73 | 0.89 | 0.0131 | 0.0700 | 0.2646 | 0.2758 | 10.3106 | 0.0319 | 4.8321 | 0.0277 |
| N51 | 0.87 | 0.64 | 0.31 | 0.27 | 0.20 | 0.30 | 0.23 | 0.85 | 0.27 | 0.40 | 0.42 | 0.17 | 0.21 | 1.01 | 0.73 | 0.65 | 0.88 | 0.0135 | 0.0587 | 0.2085 | 0.2228 | 7.7622 | 0.0249 | 3.6305 | 0.0214 |
| N43 | 0.72 | 0.66 | 0.25 | 0.34 | 0.20 | 0.29 | 0.27 | 0.93 | 0.37 | 0.39 | 0.41 | 0.18 | 0.26 | 1.02 | 0.71 | 0.71 | 0.89 | 0.0113 | 0.0458 | 0.2391 | 0.2569 | 8.0236 | 0.0259 | 2.9904 | 0.0151 |
| N11 | 0.72 | 0.68 | 0.29 | 0.44 | 0.21 | 0.26 | 0.29 | 1.11 | 0.36 | 0.28 | 0.30 | 0.30 | 0.34 | 1.04 | 0.70 | 0.71 | 0.88 | 0.0108 | 0.0517 | 0.2129 | 0.2498 | 9.0579 | 0.0260 | 4.3977 | 0.0218 |
| N29 | 1.05 | 0.60 | 0.31 | 0.26 | 0.18 | 0.28 | 0.22 | 0.93 | 0.32 | 0.44 | 0.51 | 0.30 | 0.30 | 0.93 | 0.72 | 0.71 | 0.89 | 0.0121 | 0.0603 | 0.2367 | 0.2522 | 9.8648 | 0.0273 | 4.4867 | 0.0291 |
| N1 | 0.78 | 0.48 | 0.19 | 0.19 | 0.13 | 0.22 | 0.21 | 0.84 | 0.27 | 0.30 | 0.33 | 0.16 | 0.22 | 0.75 | 0.73 | 0.68 | 0.87 | 0.0131 | 0.0723 | 0.2708 | 0.3550 | 10.6583 | 0.0302 | 4.5972 | 0.0193 |
| N50 | 0.82 | 0.65 | 0.31 | 0.36 | 0.23 | 0.33 | 0.26 | 1.02 | 0.30 | 0.42 | 0.42 | 0.19 | 0.22 | 1.16 | 0.73 | 0.66 | 0.86 | 0.0095 | 0.0406 | 0.2194 | 0.2513 | 8.5424 | 0.0226 | 3.8192 | 0.0269 |
| N3 | 0.51 | 0.63 | 0.24 | 0.31 | 0.17 | 0.33 | 0.30 | 1.25 | 0.25 | 0.26 | 0.42 | 0.21 | 0.28 | 1.00 | 0.73 | 0.70 | 0.88 | 0.0086 | 0.0720 | 0.2625 | 0.3332 | 10.0728 | 0.0216 | 4.4730 | 0.0243 |
| N15 | 0.70 | 0.57 | 0.29 | 0.33 | 0.21 | 0.30 | 0.26 | 1.00 | 0.36 | 0.45 | 0.44 | 0.15 | 0.31 | 0.93 | 0.70 | 0.71 | 0.88 | 0.0096 | 0.0533 | 0.2428 | 0.2607 | 9.6105 | 0.0267 | 4.4017 | 0.0213 |
| A23 | 0.54 | 0.61 | 0.30 | 0.37 | 0.21 | 0.32 | 0.24 | 0.96 | 0.34 | 0.47 | 0.47 | 0.18 | 0.17 | 1.11 | 0.68 | 0.63 | 0.82 | 0.0079 | 0.0774 | 0.2997 | 0.3254 | 7.6725 | 0.0297 | 4.5900 | 0.0140 |
| A3 | 0.56 | 0.60 | 0.30 | 0.27 | 0.19 | 0.28 | 0.23 | 0.85 | 0.37 | 0.42 | 0.44 | 0.17 | 0.14 | 0.96 | 0.64 | 0.60 | 0.82 | 0.0053 | 0.0744 | 0.3051 | 0.3486 | 7.0587 | 0.0362 | 4.6072 | 0.0148 |
| A18 | 0.44 | 0.65 | 0.26 | 0.38 | 0.21 | 0.27 | 0.28 | 0.92 | 0.40 | 0.44 | 0.47 | 0.21 | 0.18 | 1.01 | 0.66 | 0.64 | 0.82 | 0.0084 | 0.0450 | 0.3305 | 0.3692 | 7.3997 | 0.0341 | 4.8905 | 0.0171 |
| A10 | 0.58 | 0.69 | 0.32 | 0.35 | 0.24 | 0.27 | 0.27 | 0.90 | 0.36 | 0.48 | 0.50 | 0.19 | 0.22 | 1.09 | 0.61 | 0.62 | 0.77 | 0.0079 | 0.0759 | 0.2470 | 0.2888 | 6.9843 | 0.0325 | 4.3608 | 0.0167 |
| A24 | 0.53 | 0.67 | 0.33 | 0.42 | 0.26 | 0.30 | 0.28 | 1.03 | 0.49 | 0.51 | 0.53 | 0.22 | 0.14 | 1.23 | 0.68 | 0.64 | 0.83 | 0.0065 | 0.0606 | 0.3073 | 0.3320 | 7.8777 | 0.0318 | 4.4689 | 0.0116 |
| A8 | 0.55 | 0.63 | 0.30 | 0.33 | 0.21 | 0.25 | 0.26 | 0.82 | 0.44 | 0.45 | 0.46 | 0.19 | 0.21 | 0.99 | 0.62 | 0.62 | 0.81 | 0.0062 | 0.0670 | 0.2626 | 0.2656 | 7.7183 | 0.0324 | 5.0860 | 0.0144 |
| A25 | 0.65 | 0.69 | 0.34 | 0.32 | 0.22 | 0.36 | 0.36 | 0.91 | 0.36 | 0.49 | 0.50 | 0.17 | 0.16 | 1.10 | 0.68 | 0.63 | 0.84 | 0.0098 | 0.0821 | 0.2564 | 0.3320 | 7.5026 | 0.0326 | 4.9512 | 0.0172 |
| A26 | 0.77 | 0.83 | 0.39 | 0.37 | 0.26 | 0.38 | 0.29 | 1.10 | 0.37 | 0.57 | 0.59 | 0.22 | 0.18 | 1.32 | 0.68 | 0.62 | 0.83 | 0.0069 | 0.0813 | 0.3179 | 0.3614 | 7.2860 | 0.0364 | 4.4579 | 0.0173 |

**Supplemental Method validation**

**Method validation S1. Method validation of HPLC-UV fingerprints**

As to method validation of HPLC-UV fingerprints, precision, repeatability and stability were also confirmed for the reliability of the fingerprint method, in which RSD values of RRT and RPA of 27 peaks versus the reference peak (peak 16) were calculated. Results indicated that RSDs of RRTs and RPAs to evaluate the precision, repeatability and stability were inferior to 0.67% and 0.91%, 0.55% and 1.00%, 0.68% and 0.80% respectively.

**Method validation S2. Method validation of HPLC-ELSD fingerprints**

Method validation results of HPLC-ELSD fingerprints showed that precision, repeatability and stability were satisfied with the reasonable range. RSD values of RRT and RLPA of 4 common peaks versus the reference peak (peak 1) were calculated for evaluating precision, repeatability and stability less than 4.6% and 0.2%, 3.4% and 0.2%, 4.2% and 1.7%.
